# Vertex results for the robust analysis of uncertain biochemical systems

**DOI:** 10.1007/s00285-022-01799-z

**Published:** 2022-09-19

**Authors:** Franco Blanchini, Patrizio Colaneri, Giulia Giordano, Irene Zorzan

**Affiliations:** 1 Dipartimento di Scienze Matematiche, Informatiche e Fisiche, University of Udine, Udine, Italy; 2 Dipartimento di Elettronica, Informazione e Bioingegneria, Politecnico di Milano, Milan, Italy; 3 IEIIT-CNR, Turin, Italy; 4 Department of Industrial Engineering, University of Trento, Trento, Italy; 5 Department of Information Engineering, University of Padova, Padua, Italy; 6 Present Address: Systems Biology, School of Biosciences and Medicine, Faculty of Health and Medical Sciences, University of Surrey, Guildford, United Kingdom

**Keywords:** Biochemical systems, Robustness, Steady-state sensitivity, Vertex algorithm, 34A34, 92C40, 92C42, 92E20, 93B35, 93D09

## Abstract

We consider the problem of assessing the sensitivity of uncertain biochemical systems in the presence of input perturbations (either constant or periodic) around a stable steady state. In particular, we propose approaches for the robust sensitivity analysis of systems with uncertain parameters assumed to take values in a hyper-rectangle. We highlight vertex results, which allow us to check whether a property is satisfied for all parameter choices in the hyper-rectangle by simply checking whether it is satisfied for all parameter choices at the vertices of the hyper-rectangle. We show that, for a vast class of systems, including (bio)chemical reaction networks with mass-action kinetics, the system Jacobian has a totally multiaffine structure (namely, all minors of the Jacobian matrix are multiaffine functions of the uncertain parameters), which can be exploited to obtain several vertex results. We consider different problems: robust non-singularity; robust stability of the steady-state; robust steady-state sensitivity analysis, in the case of constant perturbations; robust frequency-response sensitivity analysis, in the presence of periodic perturbations; and robust adaptation analysis. The developed theory is then applied to gain insight into some examples of uncertain biochemical systems, including the incoherent feed-forward loop, the coherent feed-forward loop, the Brusselator oscillator and the Goldbeter oscillator.

## Introduction

In spite of inherent variations and fluctuations in the environment where they operate, leading to huge uncertainties in the parameter values of the associated models, living systems robustly preserve some crucial properties and keep reliably performing their specific task. The extraordinary robustness of biological processes has been observed in remarkable case studies, such as bacterial chemotaxis (Alon et al. 1999; Barkai and Leibler 1997) and circadian rhythms (Stelling et al. 2004). The pioneering study by Barkai and Leibler (1997), later confirmed experimentally by Alon et al. (1999), showed that the tumbling frequency that characterises the chemotaxis of *Escherichia coli* is robustly regulated: although it can rapidly change due to a variation in the concentration of a chemical stimulant, it then gradually adapts back precisely to its pre-stimulus value; this perfect adaptation property is shown to be insensitive to the precise values of the biochemical parameters, because it is a direct consequence of the network’s architecture.

The general principles underlying biological robustness have been discussed from various perspectives: Kitano (2004), Stelling et al. (2004) and Streif et al. (2016) highlight the importance of redundancy, feedback control, decoupling and modularity of cellular networks so as to ensure their robust functioning; Lesne (2008) surveys possible mechanisms that enable robust behaviours, focusing on the different meanings of robustness in the physical sciences and in the life sciences, and on the specificities of biological systems; Whitacre (2012) provides an overview of paradigms and system principles that enable biological robustness, pointing to their similarities across various levels and scales; Khammash (2016) adopts an engineering viewpoint to propose the notion of robustness as a unifying principle behind the complexity in both biological and engineered systems, and emphasises the importance of perfect adaptation (Khammash 2021) to guarantee robustness.

A variety of quantitative and numeric approaches have been used to robustly assess various properties of interest for different types of biological models with uncertain parameters. A widely investigated property is stability: Chesi (2011) has proposed methods based on optimisation and linear matrix inequalities to assess the robust stability of genetic regulatory networks including mRNAs and proteins; Waldherr and Allgöwer (2011) have leveraged polynomial programming to robustly assess stability and instability of biochemical networks; Hara et al. (2019) have applied transfer-function methods for the robust stability analysis of linear systems with generalised frequency variables to the case of cyclic gene regulatory networks. The property of concentration robustness in chemical reaction networks has been assessed by providing network-imposed sensitivity bounds to evaluate the robustness of equilibrium species concentrations against fluctuations in reactant supply (Shinar et al. 2009) and by identifying topological principles that help robustly keep the concentration of active signalling compounds within tightly defined bounds in spite of perturbations (Steuer et al. 2011), while Shinar et al. (2007) have identified a network mechanism that guarantees robust input-output relation regardless of changes in the concentration of the system components. Kim et al. (2006) and Ma and Iglesias (2002) have integrated bifurcation analysis, structural-singular-value $\mu$-analysis and hybrid optimisation to assess the robust stability of limit cycles in a model of the molecular network underlying cAMP oscillations in fields of chemotactic *Dictyostelium discoideum* cells. All these works very efficiently solve specific problems with dedicated tools.

This paper is different in spirit. Motivated by a significant class of biochemical systems and several relevant problems, we propose a framework for the robustness analysis of dynamical systems with a special uncertainty structure: *multiaffine*, i.e., affine in each uncertain parameter, while the other parameters are kept constant. Our methodologies build upon a consolidated approach to parametric robustness introduced by Barmish (1994): the main aim of the paper is to build a bridge between a class of relevant problems in mathematical biology and a class of powerful vertex results for robustness analysis developed within the realm of control theory, which offer valuable tools to assess the robustness of biological systems.

In particular, we deal with a vast class of uncertain nonlinear dynamical systems, including the interesting case of generic biochemical reaction networks, for which the uncertain parameters are assumed to take values in a hyper-rectangle and the system Jacobian is a *totally multiaffine* matrix with respect to the uncertain parameters, i.e., each minor of the Jacobian is a multiaffine function of the uncertain parameters.

Systems with totally multiaffine uncertainties include, as a special case, *BDC*-decomposable systems (Blanchini and Giordano 2014, 2017, 2019; Giordano et al. 2016), whose Jacobian matrix can be written as a linear combination of the uncertain parameters,

$$\begin{aligned} J(\delta ) = \sum _k \delta _k J_k, \end{aligned}$$

where $J_k$ are rank-one matrices: then, the Jacobian matrix can be decomposed as $J=BDC$, where *D* is a diagonal matrix carrying the uncertain parameters $\delta _k$ on the diagonal, while *B* and *C* are constant matrices such that $J_k = B_k C_k^\top$, where $B_k$ denotes the *k*th column of *B* and $C_k^\top$ the *k*th row of *C*. This class of models embraces biochemical systems with generic monotonic reaction rates, as well as general flow systems in engineering. Not only *BDC*-decomposable systems represent a proper subset of systems with totally multiaffine uncertainties: here we show that also chemical reaction networks with mass-action kinetics have a totally multiaffine Jacobian.

As a first main contribution, we provide a characterisation of totally multiaffine matrices, which allows us to simply check whether a matrix is totally multiaffine just by inspecting its entries and the matrix derivatives with respect to each of the uncertain parameters.

We then consider the steady-state sensitivity analysis for systems with totally multiaffine uncertainties. As is known, this type of analysis can be approached by considering the linearised system and computing the input-output steady-state characteristic. For systems with parametric uncertainties, the problem can be faced via robustness analysis (Barmish 1994) and solved through the solution of nonlinear systems with parametric rank-one uncertainties (Mohsenizadeh et al. 2014; Polyak and Nazin 2004). These approaches have been recently exploited to deal with the structural steady-state sensitivity analysis of *BDC*-decomposable systems affected by a persistent input perturbation, providing *vertex results* to assess the structural sign of steady-state influences (Blanchini and Giordano 2019; Giordano et al. 2016).

A vertex property is a property that holds for all parameter choices in the hyper-rectangle if and only if it holds for all parameter choices at the vertices of the hyper-rectangle. Vertex properties are therefore extremely convenient from a computational standpoint, since they can be checked for a whole continuum space (all possible realisations of the uncertain system) just by checking them at a finite set of points (the realisations associated with the vertices of the parameter space).

Here, we consider the broader class of systems with totally multiaffine uncertainties and we provide vertex results to perform their sensitivity analysis when the parameters are bounded in a hyper-rectangle.

In particular, given the totally multiaffine Jacobian $J(\delta )$, where $\delta$ is the vector stacking the uncertain parameters, each bounded in a given interval, as well as the input matrix *E* and the output matrix *H*, we present the following main results.

- Important classes of systems, including not only *BDC*-decomposable systems but also chemical reaction networks with mass-action kinetics, have a totally multiaffine uncertainty structure.
- Robust non-singularity of $J(\delta )$ can be checked by assessing its determinant at the vertices of the parameter space.
- When the system is affected by a constant perturbation, robust lower and upper bounds for the input-output sensitivity $\varSigma (\delta )=-H J(\delta )^{-1} E$ (where *H* is the output matrix and *E* is the input matrix) can be computed just by assessing $\varSigma (\delta )$ at the vertices of the parameter space; the obtained bounds are tight.
- Robust stability can be assessed resorting to the Zero Exclusion Theorem by Barmish (1994), once stability has been shown to hold for a nominal value of the parameters. To assess the stability of the nominal system, a vertex type of test (well known for systems with affine uncertainties: see the work by Garofalo et al. (1993)) can be adopted to check whether a given positive definite function, either smooth or convex, is a Lyapunov function.
- When the system is affected by a periodic perturbation, robust lower and upper bounds for the magnitude and the phase of the system transfer function $W(s,\delta )=H (sI-J(\delta ))^{-1} E$ can be computed, by leveraging vertex results.
- Potential oscillatory frequencies for the uncertain system can be robustly determined through a vertex result, which holds also in the presence of explicit delays.
- Vertex results can be obtained also to robustly assess adaptation for uncertain systems, as preliminarily reported in the conference work by Blanchini et al. (2020). When a positive persistent perturbation is applied, the output is adaptive if it initially increases and then, after a transient, it decreases so as to asymptotically recover a value that is closer to its pre-perturbation steady state. We provide a formal definition and we show that the property can be checked robustly through vertex tests.

Finally, we illustrate the proposed methodologies by applying them to chemical reaction networks, as well as biological systems taken from the literature.

## Systems with totally multiaffine uncertainties

Consider the generic nonlinear continuous-time system

$$\begin{aligned} {\left\{ \begin{array}{ll} \dot{x}(t) = f(x(t),u(t)),\\ y(t) = h(x(t)), \end{array}\right. } \end{aligned}$$

where $f :{\mathbb {R}}^n \times {\mathbb {R}} \rightarrow {\mathbb {R}}^n$ and $h :{\mathbb {R}}^n \rightarrow {\mathbb {R}}$ are continuously differentiable functions, the system state is $x(t) \in {\mathbb {R}}^n$, $u(t) \in {\mathbb {R}}$ is a scalar, which can be either an external input signal or a system parameter, and $y(t) \in {\mathbb {R}}$ is a scalar output. Assume that, given a constant input ${\bar{u}}$, the system reaches the *asymptotically stable* steady-state ${\bar{x}}$ such that $f({\bar{x}}, {\bar{u}})=0$, which corresponds to the output steady-state value ${\bar{y}} = h({\bar{x}})$, where ${\bar{x}} = {\bar{x}}({\bar{u}})$.

We wish to assess how the steady-state output ${\bar{y}} = h({{\bar{x}}})$ changes due to variations in the constant input ${\bar{u}}$. More precisely, let *v* be the perturbation affecting the input, or parameter, and denote by *w* the corresponding perturbation affecting the output. We aim to infer (or provide bounds on) the output value ${\bar{y}} + w$ corresponding to the perturbed input (or parameter) ${\bar{u}} + v$.

We consider input perturbations that are small enough to ensure that the stability of the steady state ${\bar{x}}(u)$ is preserved (recall that the eigenvalues of the Jacobian matrix are continuous functions of its entries, which are in turn continuous functions of *u*). Then, we consider the linearisation of the system at the stable equilibrium ${\bar{x}}$. Upon defining the column vector $E = \left. \frac{\partial f}{\partial u}\right| _{({\bar{x}}, {\bar{u}})}$ and the row vector $H = \left. \frac{\partial h}{\partial x}\right| _{\bar{x}}$, as well as the shifted state $z = x - {\bar{x}}$, input $v = u - {\bar{u}}$ and output $w = y - {\bar{y}}$, this boils down to considering the linearised system

1 $$\begin{aligned} {\left\{ \begin{array}{ll} \dot{z}(t) = J(\delta ) z(t) + E v(t),\\ w(t) = H z(t), \end{array}\right. } \end{aligned}$$

where $J(\delta )=\left. \frac{\partial f}{\partial x} \right| _{({\bar{x}}, {\bar{u}})}$ is the Jacobian evaluated at the equilibrium $({\bar{x}}, {\bar{u}})$ and depends on the vector of uncertain parameters $\delta = (\delta _1,\dots ,\delta _m)$. The *input-output sensitivity* (Giordano et al. 2016) is then defined as:

2 $$\begin{aligned} \varSigma = \frac{\partial w}{\partial v} = - HJ(\delta )^{-1}E. \end{aligned}$$

Although here we neglect the possible dependency of *E* and *H* on $\delta$, uncertain input and output matrices $E=E(\delta )$ and $H=H(\delta )$ can be considered under suitable assumptions; see Remark 6 in the following.

Henceforth, we consider *totally multiaffine* uncertainties: we steadily assume that the system Jacobian matrix $J(\delta )$ is totally multiaffine, a special structure that encompasses a large class of biological models.

We recall that a polynomial is multiaffine if it is affine in each variable (while the other variables are kept constant). For instance, the polynomial $p_1(\delta _1,\delta _2,\delta _3) = 2\delta _1 \delta _2 +3 \delta _2 \delta _3 - \delta _3 +4 \delta _2 +2$ is multiaffine, while $p_2(\delta _1,\delta _2) = \delta _1^2 + \delta _2 + 3$ is not multiaffine.

We assume that the Jacobian $J(\delta )$ is a polynomial matrix in $\delta = (\delta _1,\dots ,\delta _m)$ with all entries $J_{ij}(\delta )$ being multiaffine functions of $\delta = (\delta _1,\dots ,\delta _m)$. Then, we say that system (1) has totally multiaffine uncertainties if $J(\delta )$ is a totally multiaffine matrix, as now defined.

### Definition 1

*(Totally multiaffine matrix.)* Matrix $J(\delta )$ is totally multiaffine if any minor (i.e., the determinant of any square submatrix of $J(\delta )$) is a multiaffine function of $\delta = (\delta _1,\dots ,\delta _m)$. $\diamond$

### Motivation for totally multiaffine uncertainties: *BDC*-decomposable systems

A vast class of systems endowed with the property that the Jacobian matrix is totally multiaffine is represented by generic flow systems of the form

3 $$\begin{aligned} {\left\{ \begin{array}{ll} \dot{x}(t) = S g(x(t)) + E u(t),\\ y(t) = H x(t), \end{array}\right. } \end{aligned}$$

where *S* captures the topology of the flow network and the vector function *g* stacks components that are monotonic in each argument and represent flow rates. The class of systems in (3) is general enough to embrace (bio)chemical reaction networks, gene networks and metabolic networks, ecological networks and food webs, as well as compartmental systems and flow systems in engineering. In the case of a chemical reaction network (CRN), *S* is the stoichiometric matrix representing the structure of the interactions among chemical species, while the components of the vector function *g* represent reaction rates. For all systems within this class, the Jacobian matrix admits the *BDC*-decomposition introduced by Blanchini and Giordano (2014) and Giordano et al. (2016), namely it can be written as

$$\begin{aligned} J(x) = BD(x)C, \end{aligned}$$

where matrices $B \in {\mathbb {R}}^{n\times m}$, $D(x) \in {\mathbb {R}}^{m\times m}$ and $C \in {\mathbb {R}}^{m\times n}$ are constructed as follows:

*i*): *D*(*x*) is a diagonal matrix whose *m* diagonal entries are the absolute values of all the (nonzero) partial derivatives ordered as $\delta _k = |\partial g_i/\partial x_j|$, $k=1,\dots ,m$;
*ii*): the *k*th column of *B*, $B_k$, is the *i*th column of *S*;
*iii*): the *k*th row of *C*, $C_k$, has $\text{ sign }(\partial g_i/\partial x_j)$ in the *j*th position and zero elsewhere, namely, it is either the *j*th row vector of the canonical basis or its opposite, $\pm e_j^\top$.

Equivalently, *J*(*x*) can be written as the positive linear combination of rank-one matrices $B_k C_k^\top$, i.e.,

4 $$\begin{aligned} J(x)=\sum _{k=1}^m~B_k C_k^\top \delta _k(x). \end{aligned}$$

In general, the components of the vector function *g* can be considered as unknown monotonic functions, hence the diagonal entries $\delta _k$ of matrix *D* are uncertain parameters and we can see the Jacobian matrix computed at the equilibrium, $J(\delta )$, as a function of $\delta = (\delta _1,\dots ,\delta _m)$. Then, the input-output sensitivity $\varSigma$ is the ratio of two multivariate polynomials that are both multiaffine in $\delta = (\delta _1,\dots ,\delta _m)$ (Blanchini and Giordano 2019).

#### Remark 1

For a *BDC*-decomposable system, all the entries of the Jacobian matrix $J(x)=BD(x)C$ are linear functions of the diagonal elements $\delta _1,\dots ,\delta _m$ of *D*(*x*), and hence *BDC*-decomposable systems have a totally multiaffine Jacobian in view of the rank-one property, as we will see from Proposition 2. The implication is one-sided, since there exist totally multiaffine matrices that are not *BDC*-decomposable. $\diamond$

As we will see, the multiaffinity property is key to enable the efficient computation of the sensitivity $\varSigma$; this motivates us to consider a more general class of parameter-dependent matrices, totally multiaffine matrices, of which the class of *BDC*-decomposable matrices represents a proper subset.

#### Example 1

As an example of BDC-decomposable system, consider the chemical reaction networkcorresponding to the system of ordinary differential equations

$$\begin{aligned} {\left\{ \begin{array}{ll} {\dot{x}_1} = g_1 - g_{12}(x_1,x_2) - g_{13}(x_1,x_3)\\ {\dot{x}_2} = g_2 - g_{12}(x_1,x_2) \\ {\dot{x}_3} = g_{12}(x_1,x_2) - g_{13}(x_1,x_3) - g_3(x_3) \end{array}\right. } \end{aligned}$$

Its Jacobian matrix can be decomposed as

$$\begin{aligned} J = \begin{bmatrix}-(\delta _1+\delta _3) &{} -\delta _2 &{} -\delta _4 \\ -\delta _1 &{} -\delta _2 &{} 0 \\ \delta _1-\delta _3 &{} \delta _2 &{} -(\delta _4+\delta _5) \end{bmatrix} = \underbrace{\begin{bmatrix}-1 &{} -1 &{} -1 &{} -1 &{} ~0 \\ -1 &{} -1 &{} ~0 &{} ~0 &{} ~0 \\ ~1 &{} ~1 &{} -1 &{} -1 &{} -1 \end{bmatrix}}_{=B} \underbrace{\begin{bmatrix}\delta _1 &{} 0 &{} 0 &{} 0 &{} 0 \\ 0 &{} \delta _2 &{} 0 &{} 0 &{} 0 \\ 0 &{} 0 &{} \delta _3 &{} 0 &{} 0 \\ 0 &{} 0 &{} 0 &{} \delta _4 &{} 0\\ 0 &{} 0 &{} 0 &{} 0 &{} \delta _5 \end{bmatrix}}_{=D(x)} \underbrace{\begin{bmatrix}1 &{} ~0 &{} ~0\\ 0 &{} 1 &{} 0\\ 1 &{} 0 &{} 0\\ 0 &{} 0 &{} 1\\ 0 &{} 0 &{} 1\end{bmatrix}}_{=C}, \end{aligned}$$

where

$$\begin{aligned} \delta _1 := \frac{\partial g_{12}}{\partial x_1}, \quad \delta _2 := \frac{\partial g_{12}}{\partial x_2}, \quad \delta _3 := \frac{\partial g_{13}}{\partial x_1}, \quad \delta _4 := \frac{\partial g_{13}}{\partial x_3}, \quad \delta _5 := \frac{\partial g_3}{\partial x_3}, \end{aligned}$$

and is totally multiaffine in $\delta _1,\delta _2,\delta _3,\delta _4,\delta _5$. $\diamond$

### Motivation for totally multiaffine uncertainties: mass-action-kinetics systems

Another important class of systems that have a totally multiaffine Jacobian is represented by chemical reaction networks (CRNs) with mass-action kinetics. A mass-action CRN involving $n_s$ species and $n_r$ reactions is described by a system of the form

5 $$\begin{aligned} {\left\{ \begin{array}{ll} {\dot{x}}(t) = \sum _{j=1}^{n_r} S_j \kappa _j \prod _{h=1}^{n_s} x_h^{\nu _{jh}} + E u(t),\\ y(t)=H x(t), \end{array}\right. } \end{aligned}$$

where $S_j$ is the *j*th column of the stoichiometric matrix, $\kappa _j \ge 0$ is the rate of the *j*th reaction, and $\nu _{jh} \in {\mathbb {Z}}$ is the stoichiometric coefficient of the *h*th species in the *j*th reaction. Let us define the reaction rates as

$$\begin{aligned} \phi _{j} = \kappa _j \prod _{h=1}^{n_s} x_h^{\nu _{jh}}, \qquad j = 1,\dots ,n_r \end{aligned}$$

and the reciprocal variables, defined for $x_h \ne 0$, as

$$\begin{aligned} \xi _h = \frac{1}{x_h}, \qquad h = 1,\dots ,n_s. \end{aligned}$$

We can prove that a mass-action CRN has a totally multiaffine Jacobian in the reaction rates and the reciprocal variables.

#### Proposition 1

(Total multiaffinity of mass-action CRNs.) The Jacobian matrix of a mass-action CRN of the form (5) is totally multiaffine in the reaction rates and the reciprocal variables. $\square$

#### Proof

For simplicity, let us consider exclusively reactions with two reagents; the generalisation to the case of an arbitrary number of reagents is straightforward. Consider a reaction , and let $S_j$ be the column of *S* associated with this reaction. Denote as $\phi _j = \kappa _j x_i^p x_\ell ^q$ the reaction rate associated with the *j*th reaction. If, for the moment being, we do not consider the other reactions of the network, the only non-zero columns of the Jacobian *J* are the *i*th and $\ell$th ones, i.e.,

$$\begin{aligned} J&= \begin{bmatrix} \mathbf{0}_{n_s\times (i-1)}&p S_j \kappa _j x_i^{p-1} x_\ell ^q&\mathbf{0}_{n_s\times (\ell -i-1)}&q S_j \kappa _j x_i^p x_\ell ^{q-1}&\mathbf{0}_{n_s\times (n_s-\ell )} \end{bmatrix} \\&= \begin{bmatrix} \mathbf{0}_{n_s\times (i-1)}&p S_j \frac{\phi _j}{x_i}&\mathbf{0}_{n_s\times (\ell -i-1)}&q S_j \frac{\phi _j}{x_\ell }&\mathbf{0}_{n_s\times (n_s-\ell )} \end{bmatrix}\\&= \begin{bmatrix} \mathbf{0}_{n_s\times (i-1)}&p S_j \phi _j \xi _i&\mathbf{0}_{n_s\times (\ell -i-1)}&q S_j \phi _j \xi _\ell&\mathbf{0}_{n_s\times (n_s-\ell )} \end{bmatrix}\\&= \phi _j \begin{bmatrix} \mathbf{0}_{n_s\times (i-1)}&p S_j&\mathbf{0}_{n_s\times (\ell -i-1)}&q S_j&\mathbf{0}_{n_s\times (n_s-\ell )} \end{bmatrix} {{\,\mathrm{diag}\,}}(\xi _1,\dots ,\xi _{n_s})\\&\doteq \phi _j {{\mathbb {S}}}_j \varXi \end{aligned}$$

where $\varXi ={{\,\mathrm{diag}\,}}(\xi _1,\dots ,\xi _{n_s})$ is the diagonal matrix whose diagonal entries are the reciprocal variables, and ${\mathbb S}_j$ is a matrix computed from the stoichiometric matrix *S* and the stoichiometric coefficients *p*, *q*. Notice that multiplication by $\varXi$ is effective only on the *i*th and $\ell$th columns of ${{\mathbb {S}}}_j$, since all other columns of ${{\mathbb {S}}}_j$ are null. Repeating the argument for all reactions in the network, the Jacobian can be written as

$$\begin{aligned} J = \sum _{j=1}^{n_r} \phi _j {{\mathbb {S}}}_j \varXi = \left( \sum _{j=1}^{n_r} \phi _j {{\mathbb {S}}}_j \right) \varXi . \end{aligned}$$

We first show that the determinant of *J* is a multiaffine function of $\phi _1,\dots ,\phi _{n_r},\xi _1,\dots , \xi _{n_s}$. For every $j=1,\dots ,n_r$, matrix ${{\mathbb {S}}}_j$ has rank equal to 1, because all of its columns are proportional to the column vector $S_j$, i.e., ${{\mathbb {S}}}_j = S_j \mathbf{s}_j^\top$, for some row vector $\mathbf{s}_j^\top$. Then, we can resort to the matrix determinant lemma: given a square matrix *A*, column vectors *b* and *c*, and a scalar $\alpha$, $\det (A+\alpha bc^\top ) =\det (A)+ \alpha c^\top {{\,\mathrm{adj}\,}}(A) b$, where ${{\,\mathrm{adj}\,}}(A)$ is the transpose of the cofactor matrix of *A*. Exploiting the lemma, the determinant of $\left( \sum _{j=1}^{n_r} \phi _j {{\mathbb {S}}}_j \right)$ can be computed as

$$\begin{aligned} \det \left( \sum _{j=1}^{n_r} \phi _j {{\mathbb {S}}}_j \right) = \det \left( \phi _k {{\mathbb {S}}}_k + \sum _{\begin{array}{c} j=1\\ j\ne k \end{array}}^{n_r} \phi _j {{\mathbb {S}}}_j \right) = \det \left( \sum _{\begin{array}{c} j=1\\ j\ne k \end{array}}^{n_r} \phi _j {{\mathbb {S}}}_j \right) + \phi _k \mathbf{s}_k^\top {{\,\mathrm{adj}\,}}\left( \sum _{\begin{array}{c} j=1\\ j\ne k \end{array}}^{n_r} \phi _j {{\mathbb {S}}}_j \right) S_k \end{aligned}$$

and hence it is multiaffine in $\phi _1,\dots ,\phi _{n_r}$. The determinant of *J* is then given by

$$\begin{aligned} \det (J) = \det \left( \sum _{j=1}^{n_r} \phi _j {{\mathbb {S}}}_j \right) \det (\varXi ) = \det \left( \sum _{j=1}^{n_r} \phi _j {{\mathbb {S}}}_j \right) ~ \prod _{h=1}^{n_s} \xi _h, \end{aligned}$$

which is multiaffine in $\xi _1,\dots ,\xi _{n_s}$ too. Finally, to show that *J* is totally multiaffine, just note that any of its square submatrices has the form

$$\begin{aligned} {{\hat{J}}} = \left( \sum _{j=1}^{n_r} \phi _j {\hat{{\mathbb {S}}}_j} \right) {{\hat{\varXi }}} \end{aligned}$$

where ${\hat{\varXi }}$ is a diagonal matrix of appropriate size and ${\hat{{\mathbb {S}}}_j}$ is a submatrix of ${{\mathbb {S}}}_j$ having appropriate size, and hence its determinant is multiaffine in reaction rates and reciprocal variables. $\square$

#### Example 2

Consider again the CRN in Example 1, where now the reaction rate functions are assumed to follow mass-action kinetics. Define the flow variables

$$\begin{aligned} \phi _1:= g_{12}(x_1,x_2) = k_{12}x_1 x_2, \qquad \phi _2:= g_{13}(x_1,x_3) = k_{13}x_1 x_3, \qquad \phi _3:=g_3(x_3) = k_{3}x_3, \end{aligned}$$

and the reciprocal variables

$$\begin{aligned} \xi _1:=\frac{1}{x_1}, \qquad \xi _2:=\frac{1}{x_2}, \qquad \xi _3:=\frac{1}{x_3}. \end{aligned}$$

Then, the Jacobian matrix

$$\begin{aligned} J = \begin{bmatrix}-(\phi _1 + \phi _2)\xi _1 &{} -\phi _1 \xi _2 &{} -\phi _2 \xi _3 \\ -\phi _1 \xi _1 &{} -\phi _1 \xi _2 &{} 0 \\ (\phi _1 - \phi _2) \xi _1 &{} \phi _1 \xi _2 &{} -(\phi _2 + \phi _3) \xi _3 \end{bmatrix} \end{aligned}$$

is totally multiaffine in $\phi _1$, $\phi _2$, $\phi _3$, $\xi _1$, $\xi _2$, $\xi _3$. $\diamond$

#### Remark 2

Assuming uncertainty bounds on the elements of the Jacobian, rather than on the original reaction rate parameters and species concentrations, may introduce some conservatism. Consider for instance a reaction rate described by the law of mass-action as $\kappa ab$, with bounds $\kappa ^- \le \kappa \le \kappa ^+$ on the reaction rate constant, and bounds $a^- \le a \le a^+$ and $b^- \le b \le b^+$ on the species concentrations. The partial derivatives, i.e. the entries of the Jacobian, are bounded within the set

$$\begin{aligned} {\mathcal {B}} := \left\{ (\kappa a, \kappa b): \kappa ^- a^- \le \kappa a\le \kappa ^+ a^+~~~~~\text{ and }~~~~~\kappa ^- b^- \le \kappa b\le \kappa ^+ b^+ \right\} , \end{aligned}$$

and all the values in this hyper-rectangle are possible. However, since the Jacobian is evaluated at an equilibrium point, the parameters and the species concentrations are subject to the equilibrium conditions. This means that, in general, the actual set of values that the parameters and the species concentrations can take is a subset of ${\mathcal {B}}$. This is a well-known issue in parametric robustness analysis.

Conversely, no conservatism is introduced when taking the reciprocal concentrations, namely 1/*a* instead of *a*, since the former is confined exactly in $1/a^+< 1/a < 1/a^-$. Also, no conservatism is introduced when considering generic monotonic reaction rate functions, as in Example 1. Indeed, if we only know that *g*(*a*, *b*) is an increasing function of both its arguments, and we assume that, in a suitable domain, the partial derivatives are bounded as $\delta _a^- \le \partial g/\partial a\le \delta _a^+$ and $\delta _b^- \le \partial g/\partial b\le \delta _b^+$, then the equilibrium condition $g({\bar{a}}, {\bar{b}}) = {\bar{r}}$ does not introduce any restriction: any value of the derivatives within the given intervals is still possible. Even if ${\bar{r}}$ is subject to constraints, the Jacobian is not affected.

### Total multiaffinity: a general characterisation

We can provide the following characterisation of totally multiaffine matrices.

#### Proposition 2

(Characterisation of totally multiaffine matrices.) Matrix $J(\delta )$ is totally multiaffine in $\delta = (\delta _1,\dots ,\delta _m)$ if and only if each entry $J_{ij}(\delta )$ is multiaffine in $\delta$ and

$$\begin{aligned} {{\,\mathrm{rank}\,}}\left( \frac{\partial J(\delta )}{\partial \delta _k} \right) = 1, \quad \forall \, k = 1,\dots ,m. \end{aligned}$$

$$\square $$

#### Proof

*Sufficiency.* Let $M(\delta )$ be any square submatrix of $J(\delta )$. If all the entries of $J(\delta )$ are multiaffine in $\delta$, then for each $\delta _k$, $k=1,\dots ,m$, matrix $M(\delta )$ can be written as

6 $$\begin{aligned} M(\delta ) = \frac{\partial M(\delta )}{\partial \delta _k} \delta _k + {{{\tilde{M}}}}_k, \end{aligned}$$

where neither ${\partial M(\delta )}/{\partial \delta _k}$ nor ${{{\tilde{M}}}}_k$ depend on $\delta _k$. Since ${\partial M(\delta )}/{\partial \delta _k}$ has rank at most 1 by assumption, column vectors $\mathbf{m}$, $\mathbf{n}$ exist such that ${\partial M(\delta )}/{\partial \delta _k} = \mathbf{m} \mathbf{n}^\top$. Then, by applying the matrix determinant lemma, the determinant of $M(\delta )$ can be computed as

$$\begin{aligned} \det (M(\delta )) = \det ({{{\tilde{M}}}}_k) + \delta _k \mathbf{n}^\top {{\,\mathrm{adj}\,}}({{{\tilde{M}}}}_k) \mathbf{m} \end{aligned}$$

and hence it is affine in $\delta _k$. Repeating the argument for all $k=1,\dots ,m$ shows that $\det (M(\delta ))$ is multiaffine in $\delta$.

*Necessity.* Clearly, a necessary condition for $J(\delta )$ being multiaffine in $\delta$ is multiaffinity of all its entries $J_{ij}(\delta )$. Assume by contradiction that ${\partial J(\delta )}/{\partial \delta _k}$ has rank greater than 1 for some *k*. This implies that there exists a minor $M(\delta )$ of size ${{\bar{k}}} > 1$ for which ${\partial M(\delta )}/{\partial \delta _k}$ is invertible (and hence has non-zero determinant). Then, considering again (6), we have

$$\begin{aligned} \det (M(\delta )) = \det \left( \frac{\partial M(\delta )}{\partial \delta _k} \right) \, \det \left[ \delta _k I_{{\bar{k}}} + \left( \frac{\partial M(\delta )}{\partial \delta _k}\right) ^{-1} {\tilde{M}}_k \right] = \det \left( \frac{\partial M(\delta )}{\partial \delta _k} \right) p(\delta _k), \end{aligned}$$

where $p(\delta _k)=\det [\delta _k I_{{\bar{k}}} + \left( \partial M(\delta )/\partial \delta _k\right) ^{-1} {{{\tilde{M}}}}_k]$ is the characteristic polynomial, evaluated at $s=\delta _k$, of matrix $-\left( \partial M(\delta )/\partial \delta _k\right) ^{-1} {\tilde{M}}_k$, which has dimension greater than 1. Hence, $p(\delta _k)$ cannot be affine in $\delta _k$. $\square$

Proposition 2 allows us to test very simply whether a given matrix is multiaffine or not. Consider for instance the Jacobian of Example 2, whose entries are clearly multiaffine in the parameters, and compute its component-wise derivative with respect to $\phi _1$:

$$\begin{aligned} \frac{\partial J}{\partial \phi _1} = \begin{bmatrix}-\xi _1 &{} -\xi _2 &{} ~0 \\ -\xi _1 &{} -\xi _2 &{} ~0 \\ ~\xi _1 &{} ~\xi _2 &{} ~0 \end{bmatrix}. \end{aligned}$$

It is immediate to verify that $\frac{\partial J}{\partial \phi _1}$ has rank 1.

## Sensitivity analysis for totally multiaffine uncertain systems

In the following, we assume that each of the uncertain parameters $\delta _k$, $k=1,\dots ,m$, is lower and upper bounded by $\delta _k^-$ and $\delta _k^+$ respectively, i.e., $\delta _k^- \le \delta _k \le \delta _k^+$. Then, the uncertain parameter vector $\delta =(\delta _1,\dots ,\delta _m)$ belongs to the hyper-rectangle ${{\mathcal {D}}}$, which we define as:

7 $$\begin{aligned} {{\mathcal {D}}} = \left\{ \delta \in {{\mathbb {R}}}^m :\delta _k \in [\delta _k^-,\delta _k^+], k=1,\dots ,m \right\} . \end{aligned}$$

We denote by ${\hat{{\mathcal {D}}}}$ the set of vertices of the hyper-rectangle ${{\mathcal {D}}}$:

8 $$\begin{aligned} {\hat{{\mathcal {D}}}} = \left\{ {{\hat{\delta }}}\in {{\mathbb {R}}}^m :{\hat{\delta }}_k \in \left\{ \delta _k^-,\delta _k^+\right\} , k=1,\dots ,m \right\} . \end{aligned}$$

The number of vertices of ${{\mathcal {D}}}$, i.e., cardinality of the set ${\hat{{\mathcal {D}}}}$, is $2^m$: $|{\hat{{\mathcal {D}}}}| = 2^m$.

Is it possible to check whether a property holds for all possible parameters in ${\mathcal {D}}$, just by checking whether the property holds for all choices of the parameters in ${\hat{{\mathcal {D}}}}$? We denote as *vertex property* a property that is satisfied for all parameter choices in the hyper-rectangle if, and only if, it is satisfied for all parameter choices at the vertices of the hyper-rectangle: from a computational standpoint, checking a vertex property requires looking just at a finite set of parameter choices among all possible (infinite) choices.

Our first result concerns the robust non-singularity of multiaffine systems.

### Theorem 1

(Robust non-singularity.) Assume that $J(\delta )$ is totally multiaffine in $\delta$. Then, $J(\delta )$ is non-singular for all $\delta \in \mathcal{D}$ if and only if $\det \left( -J(\delta )\right)$ has the same sign (either positive or negative) for all ${{\hat{\delta }}} \in {\hat{\mathcal {D}}}$, namely on all the vertices of ${{\mathcal {D}}}$. $\square$

### Proof

*Sufficiency.* Since $J(\delta )$ is totally multiaffine, $\det (-J(\delta ))$ is a multiaffine function of $\delta$ as well. Sufficiency follows from the fact that a multiaffine function defined on a hyper-rectangle takes its maximum and minimum values at the vertices of the hyper-rectangle (see, e.g., Lemma 14.5.5 by Barmish (1994)): if the function $\det (-J(\delta ))$ is positive (or negative) at all the vertices in ${\hat{{\mathcal {D}}}}$, then it must be non-zero inside the whole hyper-rectangle ${\mathcal D}$.

*Necessity.* By contradiction, assume that the function $\det (-J(\delta ))$ takes values of opposite sign on two different vertices: $\det (-J({{\hat{\delta }}}^{(1)})) > 0$ and $\det (-J({\hat{\delta }}^{(2)}) ) < 0$, with ${{\hat{\delta }}}^{(1)}, {\hat{\delta }}^{(2)} \in {\hat{{\mathcal {D}}}}$. Then, being the determinant a continuous function of the matrix entries, there must exist some $\delta ^{(3)} \in {{\mathcal {D}}}$ such that $\det (-J(\delta ^{(3)})) = 0$. $\square$

Theorem 1 provides a vertex result that allows to easily check whether a system with totally multiaffine uncertainties is robustly non-singular. When this is the case, tight bounds on the input-output sensitivity of the system can be derived, again just by looking at the vertices of the parameter hyper-rectangle.

### Theorem 2

(Robust sensitivity bounds.) Given the linearised system (1), assume that $J(\delta )$ is totally multiaffine in $\delta$ and robustly non-singular for all $\delta \in \mathcal{D}$. Then, the input-output sensitivity $\varSigma (\delta ) = - H J(\delta )^{-1} E$ is lower and upper bounded as

$$\begin{aligned} \varSigma ^- \le \varSigma (\delta ) \le \varSigma ^+, \qquad \forall \, \delta \in {{\mathcal {D}}}, \end{aligned}$$

where

$$\begin{aligned} \varSigma ^- = \min _{{\delta } \in {\hat{{\mathcal {D}}}}} \varSigma (\delta ) \quad \text{ and } \quad \varSigma ^+ = \max _{{\delta } \in {\hat{\mathcal {D}}}} \varSigma (\delta ), \end{aligned}$$

and the bounds are tight (i.e., they are the actual minimum and maximum of $\varSigma (\delta )$). $\square$

### Proof

In view of the robust non-singularity assumption, we can write the input-output sensitivity $\varSigma (\delta )$ as

$$\begin{aligned} \varSigma (\delta ) = - H J(\delta )^{-1} E = \frac{\det \begin{bmatrix} - J(\delta ) &{} -E \\ H &{} 0 \end{bmatrix} }{\det \left( -J(\delta )\right) } = \frac{q(\delta )}{p(\delta )} \end{aligned}$$

and notice that, since $J(\delta )$ is totally multiaffine in $\delta$, both the numerator $q(\delta )$ and the denominator $p(\delta )$ are multiaffine polynomials of the parameters $\delta _1, \dots , \delta _m$. We prove the result for the lower bound $\varSigma ^-$; the proof for the upper bound $\varSigma ^+$ follows the same steps.

Assume without restriction that $p(\delta ) >0$ for all $\delta$ (if this is not the case, we can change sign to both *q* and *p*). Define the function $\rho (\delta ) = q(\delta )- k p(\delta )$, where $k \in {{\mathbb {R}}}$ is fixed. Then, $q(\delta )/p(\delta ) \ge k$ for all $\delta \in {{\mathcal {D}}}$ if and only if $\rho (\delta ) \ge 0$ for all $\delta \in {{\mathcal {D}}}$. In view of the multiaffinity of $q(\delta )$ and $p(\delta )$, the function $\rho (\delta )$ is multiaffine in $\delta$, too. Therefore, $\rho (\delta ) \ge 0$ for all $\delta \in {{\mathcal {D}}}$ if and only if $\rho (\delta ) \ge 0$ for all $\delta \in {\hat{{\mathcal {D}}}}$, which is in turn equivalent to the vertex condition $q(\delta )/p(\delta ) \ge k$ for all $\delta \in {\hat{{\mathcal {D}}}}$. Moreover, the condition holds when *k* equals the smallest value that the function $q(\delta )/p(\delta )$ takes at the vertices $\delta \in {\hat{{\mathcal {D}}}}$, i.e., $k = \varSigma ^-$, and hence the bound is tight. $\square$

### Remark 3

Assuming robust non-singularity of the system Jacobian matrix is not restrictive. In fact, since the equilibrium around which we assess the sensitivity needs to be asymptotically stable, $\det [-J(\delta )]>0$ must hold for all $\delta$, which implies robust non-singularity. Also, robust non-singularity of $J(\delta )$ is necessary for the sensitivity to be well-defined and for the result in Theorem 2 to hold: for instance, in the scalar case with $J(\delta ) = \delta _1-\delta _2$, $E=H=1$ and bounds $0.1 \le \delta _1,\delta _2 \le 1$, the resulting sensitivity $\varSigma (\delta ) = (\delta _2-\delta _1)^{-1}$ is unbounded and not even defined for $\delta _1= \delta _2$. Actually, the stability requirement could be relaxed to just requiring the non-singularity of the Jacobian at the equilibrium. Provided that the Jacobian is non-singular, we can well estimate the magnitude of the equilibrium shift under constant perturbations (by defining a steady-state map and computing its derivative) and thus perform sensitivity analysis even in the case of a possibly unstable equilibrium. $\diamond$

### Some remarks about stability analysis

Our sensitivity analysis relies on the assumption that the steady state is stable. Vertex-type approaches are available to robustly check whether such an assumption is actually satisfied.

In fact, for systems of the form (1), we can write the characteristic polynomial as

$$\begin{aligned} p(s,\delta )= \det [sI-J(\delta )] \end{aligned}$$

and check the stability of the system, for a given value $\delta ^*$, by ensuring that all the roots of $p(s,\delta ^*)$ have negative real part. Then, the Zero Exclusion Theorem by Barmish (1994) (Sect. 7.3) ensures that robust stability holds if and only if the set

$$\begin{aligned} \mathcal{V}(i \omega ) =\left\{ s=p(i \omega ,\delta ),~~\delta \in \mathcal{D} \right\} \end{aligned}$$

does not include the origin, for all frequencies $\omega \ge 0$.

Drawing this set in the complex plane is hard. However, the Mapping Theorem by Barmish (1994) (Sect. 14.6) ensures that $\mathcal{V}(i \omega )$ is in the convex hull of the vertex points $p(i \omega ,{\hat{\delta }})$, namely,

9 $$\begin{aligned} \mathcal{V}(i \omega ) \subset \text{ conv } \left\{ s=p(i\omega ,\delta ),~~\delta \in \hat{\mathcal{D}} \right\} . \end{aligned}$$

This provides a sufficient criterion for robust stability, which can therefore be checked via the following procedure:

1. check stability for an arbitrary value $\delta ^* \in {\mathcal {D}}$
2. check the exclusion for the convex hull: $0 \not \in \text{ conv } \left\{ s=p(i\omega ,\delta ),~~\delta \in \hat{\mathcal{D}} \right\}$.

The stability analysis at step 1 can be based, for instance, on Lyapunov functions: we have the following vertex result.

#### Theorem 3

(Lyapunov-based stability analysis.) Let *V*(*z*) be a positive definite radially unbounded function, which is either smooth or convex. Denote by $D^+V(z)$ the generalised Dini derivative of *V*(*z*). Then, *V*(*z*) is a Lyapunov function for system (1), in the sense that

$$\begin{aligned} D^+V(z,\delta ) \le -\beta V(z),~~~\text{ for } \text{ all }~~\delta \in \mathcal{D}, \end{aligned}$$

if and only if

$$\begin{aligned} D^+V(z, \delta ) \le -\beta V(z),~~~\text{ for } \text{ all }~~\delta \in \hat{\mathcal{D}}. \end{aligned}$$

#### Proof

Necessity follows from the fact that $\hat{{\mathcal {D}}}$ is a subset of ${\mathcal {D}}$. We now prove sufficiency. If *V*(*z*) is smooth, the proof is simple, because for any *z*

$$\begin{aligned} \psi (z, \delta ) := \nabla V(z) J(\delta ) z \end{aligned}$$

is a multilinear function of $\delta _1, \dots , \delta _q$, hence it takes its maximum value at the vertices of $\hat{\mathcal{D}}$. Therefore, denoting by $\psi ^+ (z) = \max _{\delta \in \hat{\mathcal{D}}} \psi (z, \delta )$, we have that, if $D^+V(z,\delta ) \le -\beta V(z)$ for all $\delta \in \hat{\mathcal{D}}$, then

$$\begin{aligned} D^+V(z,\delta )&= \nabla V(z) J(\delta ) z + \nabla V(z) E v\\&\le \psi ^+ (z) + \nabla V(z) E v \le -\beta V(z) ~~\text{ for } \text{ all }~~\delta \in \mathcal{D}. \end{aligned}$$

For quadratic functions the result was shown by Garofalo et al. (1993). For non-smooth but convex functions, including polyhedral Lyapunov functions (Blanchini and Giordano 2014, 2017) and piecewise-linear in rates Lyapunov functions (Al-Radawi and Angeli 1999), the proof can be carried out along the same lines, but it is more involved because one must resort to the subgradient. $\square$

## Complex sensitivity and frequency response for totally multiaffine uncertain systems

In this section, we consider the case when the perturbation acting on the input ${{\bar{u}}}$ is a periodic signal of the form $v(t) = \mu \cos (\omega t)$. Standard notions from dynamical systems theory tell us that, under robust stability assumptions (i.e., stability for all $\delta \in {\mathcal {D}}$), the steady-state output is

$$\begin{aligned} y(t) = A_\omega \mu \cos (\omega t + \phi _\omega ), \end{aligned}$$

where the magnitude amplification factor $A_\omega$ and the phase shift $\phi _\omega$ are the magnitude and phase, respectively, of the system transfer function evaluated at the perturbation frequency $\omega$.

Denote by $W(s,\delta )$ the parameter-dependent transfer function of the system:

$$\begin{aligned} W(s,\delta ) = H \left( sI - J(\delta ) \right) ^{-1} E = \frac{H {{\,\mathrm{adj}\,}}\left( sI - J(\delta ) \right) E}{\det \left( sI - J(\delta ) \right) } := \frac{q(s,\delta )}{p(s,\delta )}, \end{aligned}$$

where $q(s,\delta )$ is the numerator polynomial and $p(s,\delta )$ is the denominator polynomial corresponding to the characteristic polynomial of the system. The magnitude amplification factor $A_\omega = A_\omega (\delta )$ and the phase shift $\phi _\omega = \phi _\omega (\delta )$ are then given by

10 $$\begin{aligned} A_\omega (\delta )&= \left| H \left( i \omega I - J(\delta ) \right) ^{-1} E \right| , \end{aligned}$$

11 $$\begin{aligned} \phi _\omega (\delta )&= \angle \left( H \left( i \omega I - J(\delta ) \right) ^{-1} E \right) . \end{aligned}$$

Clearly, when the perturbation signal is constant (i.e., ${\tilde{\omega }} = 0$), the phase shift $\phi _{{{\tilde{\omega }}}}(\delta )$ is 0 for every $\delta \in {{\mathcal {D}}}$, and the amplification factor $A_{{{\tilde{\omega }}}}(\delta )$ reduces to the input-output sensitivity (i.e., $A_{{{\tilde{\omega }}}}(\delta ) = \varSigma (\delta )$), for which tight bounds are provided in Theorem 2.

We now aim to generalise the analysis of Sect. 3 (which is focused on constant input perturbations) so as to investigate how *periodic* input perturbations affect the system output.

Given a complex number $s \in {{\mathbb {C}}}$, we define ${\mathcal Q}(s)$ as the convex hull of the $2^m$ (complex) vertex points $q(s,{\delta })$, i.e.,

12 $$\begin{aligned} {{\mathcal {Q}}}(s) = {{\,\mathrm{conv}\,}}\left\{ q(s,{\delta }), ~ {\delta } \in {\hat{{\mathcal {D}}}} \right\} . \end{aligned}$$

Similarly, for the $2^m$ vertex points $p(s,{\delta })$ we denote by ${{\mathcal {P}}}(s)$ their convex hull, i.e.,

13 $$\begin{aligned} {{\mathcal {P}}}(s) = {{\,\mathrm{conv}\,}}\left\{ p(s,{\delta }), ~ {\delta } \in {\hat{{\mathcal {D}}}} \right\} . \end{aligned}$$

### Definition 2

*(Numerator/denominator critical values.)* A complex number $s \in {{\mathbb {C}}}$ is numerator critical (resp. denominator critical) if the convex polygon ${{\mathcal {Q}}}(s)$ (resp. ${{\mathcal {P}}}(s)$) includes the origin; otherwise it is numerator non-critical (resp. denominator non-critical). $\diamond$

### Proposition 3

*(Numerator/denominator non-critical values and total multiaffinity.)* Assume that $J(\delta )$ is totally multiaffine in $\delta$, and let ${{\bar{s}}} \in {{\mathbb {C}}}$ be a numerator (resp. denominator) non-critical value. Then, for any $\delta \in {\mathcal {D}}$, ${\bar{s}}$ cannot be a root of $q(s,\delta )$ (resp. $p(s,\delta )$). $\square$

### Proof

Since the convex polygon ${{\mathcal {Q}}}({\bar{s}})$ does not include the origin, there is a vector $$\begin{bmatrix}\alpha&\beta \end{bmatrix}^\top $$, with $\alpha ,\beta > 0$, that defines on the complex plane ${{\mathbb {C}}} = \{ s:s= x+iy, \, x,y\in {{\mathbb {R}}} \}$ a separation line $\alpha x + \beta y = \gamma > 0$ such that

$$\begin{aligned} \alpha x + \beta y > \gamma , \qquad \forall \, s = x + iy \in {{\mathcal {Q}}}({\bar{s}}). \end{aligned}$$

Now let $q({\bar{s}},\delta ) = \Re [q({\bar{s}},\delta )]+i \Im [q(\bar{s},\delta )]$, with $\delta \in {{\mathcal {D}}}$, where $\Re [\cdot ]$ denotes the real part and $\Im [\cdot ]$ denotes the imaginary part of a complex number. A key observation is that both $\Re [q(\bar{s},\delta )]$ and $\Im [q({\bar{s}},\delta )]$ are multiaffine functions of $\delta$. Then, consider the optimisation problem

14 $$\begin{aligned} \min _{\delta \in {{\mathcal {D}}}} ~ \alpha \Re [q({\bar{s}},\delta )] + \beta \Im [q({\bar{s}},\delta )] = \min _{{\delta } \in {\hat{\mathcal {D}}}} ~\alpha \Re [q({\bar{s}},{\delta })] + \beta \Im [q(\bar{s},{\delta })] > \gamma , \end{aligned}$$

where the equality holds because the objective function is multiaffine in $\delta$, and hence takes its minimum in the vertex set $\hat{{\mathcal {D}}}$, while the inequality is due to the fact that all points $q({{\bar{s}}},{\delta })$ belong to ${{\mathcal {Q}}}(\bar{s})$. Since $\gamma >0$, $q({{\bar{s}}},\delta )\ne 0$ for all $\delta \in {{\mathcal {D}}}$, namely $q(s,\delta )$ cannot have a root at $\bar{s}$. The proof for $p(s,\delta )$ is identical. $\square$

### Remark 4

Proposition 3 restates well-known results about value-set analysis: the proof is reported for completeness, yet a different proof could be given in terms of the Mapping Theorem by Barmish (1994) (Section 14.6). $\diamond$

We separately consider the numerator and denominator polynomials, $q(s,\delta )$ and $p(s,\delta )$, and provide bounds on their magnitude and phase by exploiting the fact that they are multiaffine functions of $\delta$. Again, we can exploit vertex results.

### Theorem 4

(Magnitude and phase of numerator/denominator polynomials.) Assume that $J(\delta )$ is totally multiaffine in $\delta$ and let ${{\bar{s}}} \in {{\mathbb {C}}}$ be a numerator non-critical value. Then, for every $\delta \in {{\mathcal {D}}}$, the following inequalities hold:

$$\begin{aligned} \angle q({\bar{s}},\delta ) \,&\, \le \theta ^+_q \doteq \max _{{\delta } \in {\hat{{\mathcal {D}}}}} \angle q({\bar{s}}, {\delta }),\\ \angle q({\bar{s}},\delta ) \,&\, \ge \theta ^-_q \doteq \min _{{\delta } \in {\hat{{\mathcal {D}}}}} \angle q({\bar{s}},{\delta }),\\ |q({\bar{s}},\delta )| \,&\, \le \mu ^+_q \doteq \max _{{\delta } \in {\hat{{\mathcal {D}}}}} |q({\bar{s}},{\delta })|,\\ |q({\bar{s}},\delta )| \,&\, \ge \mu ^-_q \doteq \min _{\sigma \in {{\mathcal {Q}}}({\bar{s}})} \Vert \sigma \Vert . \end{aligned}$$

The first three bounds are tight, while the last, computed as the minimum Euclidean norm of the points in ${{\mathcal {Q}}}({\bar{s}})$, is conservative in general. If ${{\bar{s}}} \in {{\mathbb {C}}}$ is a denominator non-critical value, then analogous bounds hold for $p(s,\delta )$. $\square$

### Proof

Recall that, since $J(\delta )$ is totally multiaffine in $\delta$, the numerator $q(s,\delta )$ of the transfer function is multiaffine in $\delta$ too (and the same holds true for the denominator $p(s,\delta )$). The thesis then follows as an application of the Mapping Theorem by Barmish (1994) (Section 14.6), which guarantees that the convex hull of the set of $q({{\bar{s}}},\delta )$, $\delta \in {{\mathcal {D}}}$, is the convex hull of the vertex points $q({{\bar{s}}},{\delta })$, ${\delta } \in {\hat{{\mathcal {D}}}}$, i.e.,

$$\begin{aligned} {{\,\mathrm{conv}\,}}\left\{ q({{\bar{s}}},\delta ), ~ \delta \in {{\mathcal {D}}} \right\} = {{\,\mathrm{conv}\,}}\left\{ q({\bar{s}},{\delta }), ~ {\delta } \in {\hat{\mathcal {D}}} \right\} = {{\mathcal {Q}}}({{\bar{s}}}). \end{aligned}$$

**Fig. 1:**
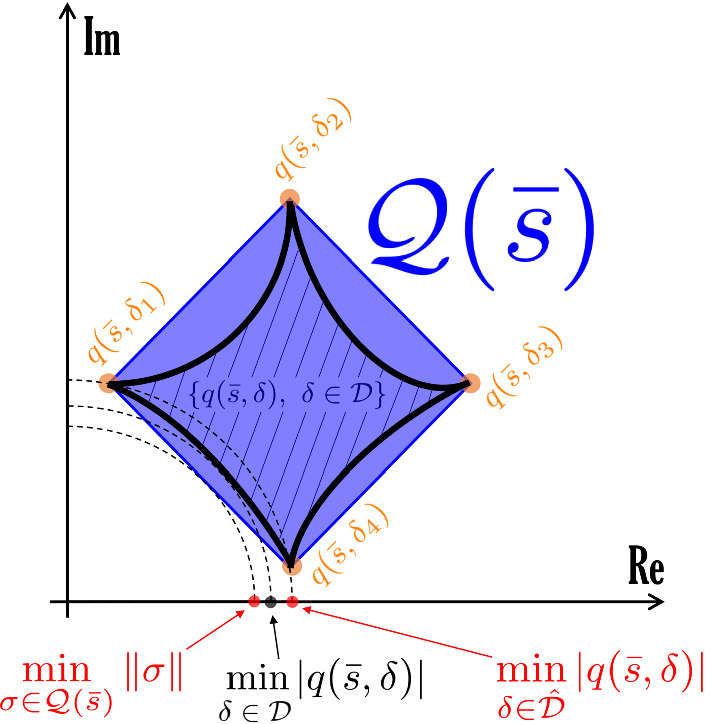
Example of a set $\{q({\bar{s}}, \delta ), \, \delta \in {\mathcal {D}} \}$ (drawn with a thick black boundary and filled with diagonal lines) and its convex hull ${{\mathcal {Q}}}({\bar{s}}) = {{\,\mathrm{conv}\,}}\left\{ q(s,{\delta }), ~ {\delta } \in {\hat{{\mathcal {D}}}}= \{\delta _1,\delta _2,\delta _3,\delta _4\} \right\}$ (represented as a blue square), where $\delta _1$, $\delta _2$, $\delta _3$,$\delta _4$ are the vertices of the hyper-rectangle ${\mathcal {D}}$. Note that the minimum modulus of $q({\bar{s}}, \delta )$ for $\delta \in {\mathcal {D}}$ is smaller than the modulus at the vertices: this justifies the lower bound in Theorem 4, which is possibly conservative, as in this case. Indeed, $\min _{\sigma \in {\mathcal Q}({\bar{s}})} \Vert \sigma \Vert \le \min _{\delta \in {\mathcal {D}}} |q(\bar{s}, \delta )| \le \min _{{\delta } \in {\hat{{\mathcal {D}}}}} |q(\bar{s},{\delta })|$ (color figure online)

In particular, the minimum and maximum values of the phase of $q({\bar{s}}, \delta )$, for $\delta \in {{\mathcal {D}}}$, are obtained at the vertices, for some $\delta \in {\hat{{\mathcal {D}}}}$. The same holds true for the maximum value of the modulus of $q({\bar{s}}, \delta )$. The minimum value of the modulus of $q({\bar{s}}, \delta )$, for $\delta \in {{\mathcal {D}}}$, is lower bounded by the minimum value in the convex hull, $\min _{\sigma \in {{\mathcal {Q}}}({\bar{s}})} \Vert \sigma \Vert$, which is in general smaller or equal to $\min _{{\delta } \in {\hat{{\mathcal {D}}}}} |q({\bar{s}},{\delta })|$; see, for instance, the example in Fig. 1. However, this fourth bound is not tight: as shown in the example in Fig. 1, the true minimum $\min _{\delta \in {\mathcal {D}}} |q({\bar{s}}, \delta )|$ is between these two values: $\min _{\sigma \in {{\mathcal {Q}}}({\bar{s}})} \Vert \sigma \Vert \le \min _{\delta \in {\mathcal {D}}} |q({\bar{s}}, \delta )| \le \min _{{\delta } \in {\hat{{\mathcal {D}}}}} |q({\bar{s}},{\delta })|$. $\square$

We are now ready to evaluate magnitude and phase of the transfer function, as an immediate consequence of Theorem 4.

### Theorem 5

(Bounds on magnitude and phase of the transfer function.) Assume that $J(\delta )$ is totally multiaffine in $\delta$ and let $i \omega$ be both numerator non-critical and denominator non-critical. Then, for every $\delta \in {{\mathcal {D}}}$, the following inequalities hold:

15 $$\begin{aligned} \frac{\mu ^-_q}{\mu ^+_p} \,&\le \, A_\omega (\delta ) \, \le \, \frac{\mu ^+_q}{\mu ^-_p}, \end{aligned}$$

16 $$\begin{aligned} \theta ^-_q - \theta ^+_p \,&\le \, \phi _\omega (\delta ) \, \le \, \theta ^+_q - \theta ^-_p. \end{aligned}$$

$$\square $$

### Remark 5

Bounds (15)-(16) are in general conservative. The only tight bounds are those achieved on the phase shift $\phi _\omega (\delta )$ when the numerator is a constant. We point out that these bounds are valid for small enough input signals: in this case, the linear approximation is valid and our results transfer to the original nonlinear system. However, for large signals, the prediction capability could be lost. For instance, large signals could drive some system components into saturations and, in this case, the resulting amplitude and phase could be different from those estimated by our analysis. $\diamond$

### Bounds on magnitude and phase plots of the transfer function

The tools developed in this section allow us to derive bounds on the Bode plot of the system transfer function, which represents the magnitude and the phase of the transfer function $W(i\omega ,\delta )$ as a function of the frequency $\omega$. More precisely, plotting the bounds (15) and (16) as a function of the perturbation frequency $\omega$ provides the admissible intervals for magnitude and phase plots, respectively. Since these bounds are not tight, we introduce the concept of inner and outer bounds to assess how conservative they are.

#### Definition 3

*(Outer bounds for magnitude and phase.)* Functions $A^-_{out}(\omega )$ and $A^+_{out}(\omega )$ are *outer lower and upper bounds* for the output magnitude amplification factor $A_\omega (\delta )$ if, for every $\omega \ge 0$, it holds

17 $$\begin{aligned} A^-_{out}(\omega ) \, \le \, A_\omega (\delta ) \, \le \, A^+_{out}(\omega ), \qquad \forall \, \delta \in {{\mathcal {D}}}. \end{aligned}$$

Functions $\phi ^-_{out}(\omega )$ and $\phi ^+_{out}(\omega )$ are *outer* lower and upper bounds for the output phase shift $\phi _\omega (\delta )$ if, for every $\omega \ge 0$, it holds:

18 $$\begin{aligned} \phi ^-_{out}(\omega ) \, \le \, \phi _\omega (\delta ) \, \le \, \phi ^+_{out}(\omega ), \qquad \forall \, \delta \in {{\mathcal {D}}}. \end{aligned}$$

$$\diamond $$

Clearly, the bounds in (15) and (16) intended as functions of the frequency $\omega$, i.e.,

$$\begin{aligned} A^-_{out}(\omega ) = \frac{\mu ^-_q(\omega )}{\mu ^+_p(\omega )}, \qquad A^+_{out}(\omega ) = \frac{\mu ^+_q(\omega )}{\mu ^-_p(\omega )} \end{aligned}$$

and

$$\begin{aligned} \quad \phi ^-_{out}(\omega ) =\theta ^-_q(\omega ) - \theta ^+_p(\omega ), \qquad \phi ^+_{out}(\omega ) = \theta ^+_q(\omega ) - \theta ^-_p(\omega ), \end{aligned}$$

are outer lower and upper bounds for the output magnitude amplification factor and the output phase shift, respectively. As already remarked, these intervals are in general conservative. To evaluate the degree of conservatism, we can introduce *inner lower and upper bounds*, so as to derive sub-intervals that are necessarily included in the outer intervals, as per the following vertex result.

#### Proposition 4

(Inner bounds for magnitude and phase.) Assume that $J(\delta )$ is totally multiaffine in $\delta$ and introduce the following functions of the frequency $\omega \ge 0$:

$$\begin{aligned} A^-_{in}(\omega )&= \min _{{\delta } \in {\hat{{\mathcal {D}}}}} A_\omega (\delta ),&A^+_{in}(\omega )&= \max _{{\delta } \in {\hat{{\mathcal {D}}}}} A_\omega (\delta ),\\ \phi ^-_{in}(\omega )&= \min _{{\delta } \in {\hat{{\mathcal {D}}}}} \phi _\omega (\delta ),&\phi ^+_{in}(\omega )&= \max _{{\delta } \in {\hat{{\mathcal {D}}}}} \phi _\omega (\delta ), \end{aligned}$$

where $A_\omega (\delta )$ and $\phi _\omega (\delta )$ are defined as in (10) and (11), respectively. Then, for every $\omega \ge 0$ and for every $\delta \in {\mathcal D}$, the following inclusions hold:

$$\begin{aligned} \left[ A^-_{in}(\omega ), A^+_{in}(\omega ) \right]&\subseteq \left[ A^-_{out}(\omega ), A^+_{out}(\omega )\right] \\ \left[ \phi ^-_{in}(\omega ), \phi ^+_{in}(\omega )\right]&\subseteq \left[ \phi ^-_{out}(\omega ), \phi ^+_{out}(\omega )\right] \end{aligned}$$

$$\square $$

#### Proof

The values $A^-_{in}(\omega )$, $A^+_{in}(\omega )$, $\phi ^-_{in}(\omega )$ and $\phi ^+_{in}(\omega )$ are actually achieved for some vertex ${\delta } \in {\hat{{\mathcal {D}}}}$, hence the corresponding bounds cannot be tightened. Therefore, the inclusions $[ A^-_{in}(\omega ), A^+_{in}(\omega )] \subseteq [ A^-_{out}(\omega ), A^+_{out}(\omega )]$ and $[ \phi ^-_{in}(\omega ), \phi ^+_{in}(\omega )] \subseteq [ \phi ^-_{out}(\omega ), \phi ^+_{out}(\omega )]$ hold. $\square$

#### Remark 6

All the results we have presented so far can be extended to the case in which $E = \left. \frac{\partial f}{\partial u}\right| _{({\bar{x}}, {\bar{u}})}$ and $H = \left. \frac{\partial h}{\partial x}\right| _{{\bar{x}}}$ are also uncertain, provided that the matrix

$$\begin{aligned} \begin{bmatrix} -J(\delta ) &{} -E(\delta ) \\ H(\delta ) &{} 0 \end{bmatrix} \end{aligned}$$

is totally multiaffine in $\delta$. Then, the transfer function can be written as

$$\begin{aligned} {{{\tilde{W}}}}(s,\delta ) = H(\delta )(s I - J(\delta ))^{-1}E(\delta ) = \frac{\det \begin{bmatrix} s I - J(\delta ) &{} -E(\delta )\\ H(\delta ) &{} 0 \end{bmatrix} }{\det (s I - J(\delta ))} = \frac{{\tilde{q}}(s,\delta )}{{\tilde{p}}(s,\delta )} \end{aligned}$$

and Theorem 5 holds true without changes, as well as all the results in Sect. 4. Also all the results in Sect. 3 remain valid: in fact, the sensitivity in Theorem 2 corresponds to the transfer function computed at $s=0$. $\diamond$

### Self-sustained oscillations: what do the critical frequencies tell us?

Critical frequencies are fundamental when analysing oscillators. If, for a certain value of the parameters, an equilibrium point becomes marginally stable with two complex eigenvalues on the imaginary axis, the imaginary part of these eigenvalues is expected to provide a good estimate of the oscillating frequency $\omega$. This reasoning is heuristic in the following sense. The onset of oscillations requires the presence of a negative loop with sufficiently high loop gain (see, e.g., the work by Blanchini et al. 2014; Domijan and Pécou 2011; Gouze 1998; Snoussi 1998 and the references therein). If all the eigenvalues lie to the left of the imaginary axis, oscillations cannot be persistent. If the eigenvalues are exactly on the imaginary axis, the oscillations of the linear (linearised) systems are persistent, but, if these eigenvalues cross the imaginary axis (even slightly, due to a perturbation), then the oscillatory trajectories diverge in principle. However, unavoidable saturations in the original nonlinear system cause a “virtual gain reduction", which keeps the oscillations bounded. This situation can be seen as due to the reduction of the *critical gain*, namely the gain value corresponding to purely imaginary eigenvalues. An approximate guess of the oscillating frequency can therefore be obtained as follows.

#### Definition 4

(Potential oscillatory frequency.) The value $\omega$ is a *potential oscillatory frequency* if $p(i \omega ,\delta )=0$ for some $\delta \in {{\mathcal {D}}}$. $\diamond$

The next vertex result, a corollary to Proposition 3, allows us to identify potential oscillatory frequencies.

#### Theorem 6

(Potential oscillatory frequencies and denominator critical values.) A potential oscillatory frequency $\omega$ must be such that ${\bar{s}} = i \omega$ is a denominator critical value as per Definition 2, namely, the convex polygon ${\mathcal {P}}({\bar{s}})={{\,\mathrm{conv}\,}}\{p({\bar{s}}, \delta ), \delta \in {\hat{{\mathcal {D}}}}\}$ includes the origin. $\square$

The opposite is not true in general: if $i\omega$ is a denominator critical value, it still could be that no parameter value exists in the admissible bounds for which there are imaginary roots. In fact, the convex hull ${\mathcal {P}}({\bar{s}})={{\,\mathrm{conv}\,}}\{p({\bar{s}}, \delta ), \delta \in {\hat{{\mathcal {D}}}}\}$ is larger than the actual set $\{p({\bar{s}}, \delta ), \delta \in {{\mathcal {D}}}\}$, and it may happen that $0 \in {\mathcal {P}}({\bar{s}})$ but $0 \not \in \{p({\bar{s}}, \delta ), \delta \in {{\mathcal {D}}}\}$. Still, the polygon ${\mathcal {P}}(\bar{s})$ provides good indications about the oscillation frequency we can expect.

The previous considerations can be applied also to the case of a stable system with a negative loop including delays that cause the onset of oscillations.

Consider the system with $y(s)=W(s)u(s)$ and a delay $\tau$ in the closed loop, $u(t)=y(t-\tau )$, hence $u(s)=e^{-s\tau }y(s)$. To analyse its oscillatory behaviour, we need to study the quasi-polynomial

$$\begin{aligned} \varPsi (s,\tau ,\delta ) =q(s,\delta ) e^{-s\tau } + p(s,\delta ) \end{aligned}$$

and the existence of possible roots in $i \omega$. For any fixed $\omega$, the set of all $\varPsi (i \omega ,\tau ,\delta )$ enjoys the same property of the polynomials $q(s,\delta )$ and $p(s,\delta )$: the convex hull of the set is the convex hull of the vertices. Therefore, the previous analysis holds unchanged.

## Robust adaptation analysis for uncertain systems

In this section, we report vertex results to assess *adaptation* to a persistent constant input (step input), a remarkable property of several biological systems (Alon 2006; Ma et al. 2009). An input-output dynamical system exhibits adaptation if, after a persistent input has been applied, the output initially increases and, after a transient, eventually decreases so as to get close(r) to its pre-perturbation value. Adaptation includes *perfect adaptation* (El-Samad et al. 2002; Giordano et al. 2016; Khammash 2021; Kim et al. 2014; Yi et al. 2000), which is achieved when the output asymptotically recovers exactly its pre-perturbation value and can be formally associated with the system transfer function *W*(*s*) vanishing for $s=0$.

**Fig. 2:**
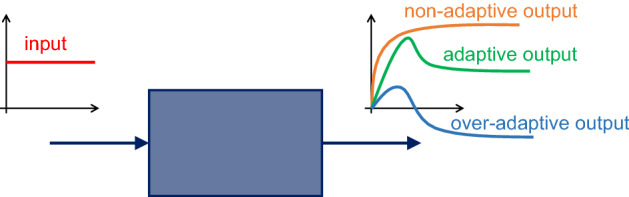
System with non-adaptive (orange), adaptive (green) and over-adaptive (blue) responses to a step input (red) (color figure online)

Adaptation is difficult to define formally and then to assess, even more so in the case of systems with uncertain parameters; see the discussion by Blanchini et al. (2020). It roughly requires the step response to be (essentially) first increasing and then decreasing: temporary trend changes and oscillations can occur during the transient, but the response must be *predominantly* decreasing for large values of *t*. For instance, consider the three output signals shown in Fig. 2 in response to a step perturbation (red signal): we call the orange one, which does not eventually decrease, non-adaptive; the green one, which first increases and then decreases, adaptive; the blue one, which first increases and then decreases so much that it changes sign, over-adaptive.

Throughout this section, we consider the following standing assumption.

### Assumption 1

Consider system (1), with $\delta \in {\mathcal {D}}$. The step response $y_s(t)$ corresponding to the input $u(t) = {\bar{u}}$ is initially positive (i.e. $\exists ~\tau >0$ such that $\dot{y}_s(t)\doteq \frac{d}{dt}y_s(t)>0$ for $0 < t \le \tau$) and $\lim _{t \rightarrow \infty } y_s(t)$ is finite. $\diamond$

The sign of ${\bar{u}}$ is therefore implicitly chosen to ensure that $y_s(t)$ is initially positive. We can say that the system is adaptive if $y_s(t)$ is essentially increasing for small values of *t* and essentially decreasing for larger values of *t*. In order to provide a formal definition, we consider the weighted integral

19 $$\begin{aligned} I_a = \int _0^\infty e^{at} \dot{y}_s(t) dt. \end{aligned}$$

Then, the abscissa of convergence, $\sigma$, is the largest value of *a* for which the integral in (19) is finite:

20 $$\begin{aligned} \sigma = \sup \{a: |I_a| < \infty \}. \end{aligned}$$

Note that, when $a=0$, the integral in (19) is equal to $\lim _{t \rightarrow \infty } y_s(t)$ in view of the Final Value Theorem, hence it is finite according to Assumption 1. We can then provide a formal definition of adaptation (which does not require linearity of the system in general; see Blanchini et al. 2020).

### Definition 5

*(Adaptive step response.)* Given the weighted integral $I_a$ defined in (19), the response $y_s(t)$ to the step input ${\bar{u}}$, starting from zero initial conditions, is *adaptive* if there exists ${\bar{a}}< \sigma$ such that

$$\begin{aligned}\begin{array}{ll} I_a > 0&{} \text{ for }~~ a< {\bar{a}},\\ I_a< 0&{}\text{ for }~~ {\bar{a}}< a < \sigma . \end{array} \end{aligned}$$

More precisely, we say it is *partially adaptive* if ${\bar{a}} >0$; *perfectly adaptive* if ${\bar{a}} =0$; *over-adaptive* if ${\bar{a}} <0$. Given the uncertain system (1) with step response $y_s(t,\delta )$, adaptation is *robust* if the property holds for all ${\bar{u}}$ and all $\delta \in {\mathcal {D}}$. $\diamond$

The idea is that, when $a<0$, the values of $\dot{y}_s(t)$ at earlier times (mainly positive in an adaptive system) are weighed more, while when $a>0$ the values of $\dot{y}_s(t)$ at later times (mainly negative in an adaptive system) are weighed more; the larger is *a* in absolute value, the more visible such an effect is.

Perfect adaptation corresponds to $\dot{y}_s(t)$ having zero mean on the interval $[0,\infty )$, i.e., $\lim _{t \rightarrow \infty } y_s(t)=I_0=0$. Partial adaptation corresponds to $\dot{y}_s(t)$ having zero mean on the interval $[0,\infty )$ if weighted by an *increasing* exponential $e^{{{\bar{a}}}t}$ (${\bar{a}}>0$); namely, $\lim _{t \rightarrow \infty } y_s(t)=I_0>0$. Over-adaptation corresponds to $\dot{y}_s(t)$ having zero mean on the interval $[0,\infty )$ if weighted by a *decreasing* exponential $e^{{{\bar{a}}}t}$ (${\bar{a}} <0$); namely, $\lim _{t \rightarrow \infty } y_s(t)=I_0<0$. Finally, sign-definiteness of $\dot{y}_s(t)$ (either positive or negative) implies the lack of adaptation (but the converse is not true: there are non-adaptive systems for which $\dot{y}_s(t)$ is not sign-definite).

Consider system (1) with impulse response $\dot{y}_s(t)$, namely, the rational transfer function *W*(*s*) is the Laplace transform of $\dot{y}_s(t)$ for ${\bar{u}}=1$. Then, the abscissa of convergence of *W*(*s*) is $\sigma$, defined in (20), while $-\sigma$ is the spectral abscissa, namely, the largest among the real parts of the poles of *W*(*s*) (recall that the poles of a transfer function are the roots of its denominator polynomial). Given the complex numbers $w_1$ and $w_2$, we say that $w_1$ *dominates* $w_2$ if $\Re (w_1) > \Re (w_2)$. Then, the following proposition characterises adaptation as the presence of a single dominant real zero, larger than the spectral abscissa.

### Proposition 5

(Characterisation of adaptive step response.) Let $W(s)=H(sI-J)^{-1}E=q(s)/p(s)$ be the rational, strictly proper transfer function of the linear asymptotically stable system (1). The system step response is adaptive if and only if there exists exactly one real zero, $-\zeta$, such that $\zeta < \sigma$. Partial adaptation corresponds to $\zeta >0$, perfect adaptation to $\zeta =0$, over-adaptation to $\zeta <0$. $\square$

### Proof

When $a<\sigma$, $I_a = \int _0^\infty e^{at}\dot{y}_s(t) dt = \lim _{s \rightarrow 0} W(s-a) = W(-a)$. Then, the threshold value is $\bar{a}=\zeta$, where $-\zeta$ is a real zero, which must be the only zero in the open interval $(-\sigma ,\infty )$ in order to prevent other sign changes. $\square$

Now, given the uncertain system (1), we wish to check whether it has a *robustly adaptive* response. To this aim, we consider the system transfer function $W(s,\delta )=H(sI-J(\delta ))^{-1}E=q(s,\delta )/p(s,\delta )$. Then, for all admissible values of $\delta$, we evaluate both the position of its real zeros (real roots of $q(s,\delta )$) and the spectral abscissa $-\sigma$ (i.e., the largest real part of all poles, namely, of all roots of $p(s,\delta )$). If, for all possible values of the uncertain parameters, there is a single real zero $-\zeta$ with $\zeta <\sigma$, this reveals *robust adaptation*.

We approach this problem through vertex algorithms and graphical methods based on the theory of parametric robustness and the Mapping Theorem by Barmish (1994).

In particular, the spectral abscissa $-\sigma$ can be robustly evaluated as follows.

### Proposition 6

(Robust spectral abscissa.) The value $-a$ is a *robust spectral abscissa*, namely, $a < \sigma (\delta )$ for all $\delta \in {\mathcal {D}}$, if

$$\begin{aligned} 0 \not \in {\mathcal {P}}(i \omega - a),~~~\forall \omega \ge 0, \end{aligned}$$

with ${\mathcal {P}}(i \omega - a)$ defined as in (13). $\square$

In fact, the spectral abscissa $-\sigma$ is less than $-a$ if and only if all the roots of the characteristic polynomial $p(s,\delta )$ have real parts less than $-a$, which is equivalent to $p(s-a,\delta )$ being Hurwitz for all $\delta \in {\mathcal {D}}$. Since the convex hull of $\{p(s-a,\delta ), \, \delta \in {\mathcal {D}}\}$ is equal to ${\mathcal {P}}(i \omega - a)$, the convex hull of $\{p(s-a,\delta ), \, \delta \in {\hat{{\mathcal {D}}}}\}$, the proposition provides a sufficient condition. For a fixed *a*, the condition can be checked in practice by plotting via computer graphics the set ${\mathcal {P}}(i \omega - a)$ for a finite frequency range, sufficiently large and sufficiently densely sampled.

As an alternative, one can also check whether $p(s-a,\delta )$ is Hurwitz through the solution of a convex optimisation problem, as done by Blanchini et al. (2019): this approach is harder to implement, but it is non-conservative.

The position of the real zeros and poles can be robustly evaluated based on a vertex result.

### Proposition 7

(Characterisation of zeros and poles.) The real $\lambda$ is a zero of the transfer function $W(s,\delta )$, i.e. $q(\lambda ,\delta ) = 0$, for some $\delta \in {\mathcal {D}}$ if and only if functions

$$\begin{aligned} \varphi ^-(\lambda )=\min \left\{ q(\lambda ,\delta ),~~ \delta \in {\hat{\mathcal {D}}} \right\} \qquad \text{ and } \qquad \varphi ^+(\lambda )=\max \left\{ q(\lambda , \delta ), ~~\delta \in {\hat{\mathcal {D}}} \right\} \end{aligned}$$

have opposite sign.

Analogously, the real $\lambda$ is a pole of the transfer function $W(s,\delta )$, i.e. $p(\lambda ,\delta ) = 0$, for some $\delta \in {\mathcal {D}}$ if and only if functions

$$\begin{aligned} \psi ^-(\lambda )= \min \left\{ p(\lambda , \delta ), ~~ \delta \in {\hat{\mathcal {D}}} \right\} \qquad \text{ and } \qquad \psi ^+(\lambda )= \max \left\{ p(\lambda , \delta ),~~ \delta \in {\hat{\mathcal {D}}} \right\} \end{aligned}$$

have opposite sign.

Functions $\varphi ^-(\lambda )$ and $\varphi ^+(\lambda )$ (resp. $\psi ^-(\lambda )$ and $\psi ^+(\lambda )$) provide tight bounds, namely, for each real $\lambda$ they provide the actual minimum and maximum value of $q(\lambda ,\delta )$ (resp. $p(\lambda ,\delta )$) over $\delta \in {{\mathcal {D}}}$. $\square$

### Proof

Given a fixed $\lambda$, the polynomial $q(\lambda ,\delta )$ is multiaffine in $\delta$, so it does achieve both its minimum, $\varphi ^-(\lambda )$, and its maximum, $\varphi ^+(\lambda )$, on some vertex of the hyper-rectangle ${\mathcal {D}}$. Therefore, if $\varphi ^-(\lambda )$ and $\varphi ^+(\lambda )$ have the same sign, also $q(\lambda ,\delta )$ has the same sign. Conversely, if these extrema have different sign, i.e. $\varphi ^-(\lambda )\! \le \! 0 \! \le \! \varphi ^+(\lambda )$, then continuity implies the existence of some $\delta ^*$ such that $q(\lambda ,\delta ^*)=0$. The proof for the pole case is identical. $\square$

The previous result entails that we can draw the exact envelope of the real plot of the polynomials $p(\lambda ,\delta )$ and $q(\lambda ,\delta )$ as a function of $\lambda$, which we call *robust real plot*. Drawing the two functions $\varphi ^-(\lambda )$ and $\varphi ^+(\lambda )$ (resp. $\psi ^-(\lambda )$ and $\psi ^+(\lambda )$) is enough to locate the real zeros (resp. real poles) of the uncertain system.

In order to assess robust adaptation for an uncertain system, we need to check whether the system has a real dominant zero for all possible values of the uncertain parameters. First of all, we need to verify that Assumption 1 holds robustly: this can be done through a vertex test.

### Lemma 1

(Robust vertex check.) Assumption 1 holds for all $\delta \in {\mathcal {D}}$ if and only if the leading coefficient of $q(s,\delta )$ is positive for all $\delta \in {\hat{\mathcal {D}}}$. $\square$

### Proof

The step response is positive in a right neighbourhood of 0 due to Assumption 1. Hence, the first non-zero derivative of $\dot{y}_s(t)$, of order *r* (relative degree of $W(s,\delta )$, namely, difference between the degree of the denominator and the degree of the numerator), must be positive. The initial value theorem yields $\lim _{t\rightarrow 0} y_s^{(r)}(t) = \lim _{s\rightarrow \infty } s^r W(s,\delta ) > 0$, hence the leading coefficient $q_{n-r}(\delta )$ of $q(s,\delta )$ must be positive for all $\delta \in {\mathcal {D}}$. Since $q_{n-r}(\delta )$ is a multiaffine function, its minimum is achieved at the vertices $\delta \in {\hat{\mathcal {D}}}$. Then, it is necessary and sufficient that all the values at the vertices are positive. $\square$

### Definition 6

(Robust real zero dominance.) If $-\sigma <0$ is a robust spectral abscissa (i.e., it dominates all poles for all $\delta \in {\mathcal {D}}$ ), the system with transfer function $W(s,\delta )$ is *robustly real zero dominant* if, for all $\delta \in {\mathcal {D}}$, there exists a real zero that dominates $-\sigma$. $\diamond$

Robust real zero dominance can be checked through a vertex result.

### Proposition 8

(Checking robust real zero dominance.) Let $-\sigma$ be a robust spectral abscissa for the transfer function $W(s,\delta )$, $\delta \in {\mathcal {D}}$. Then the system has the robust real zero dominance property if $\varphi ^+(-\sigma ) < 0$. Furthermore, the real dominant zero is unique if $q'(\lambda ,\delta )$, the derivative of $q(\lambda ,\delta )$ with respect to $\lambda$, is positive for all ${\hat{\delta }} \in {\hat{\mathcal {D}}}$ and for all $\lambda \in [-\sigma , \infty )$. $\square$

### Proof

The leading coefficient of $q(s,\delta )$ is positive under Assumption 1, hence $q(\lambda ,\delta )$ converges to $+\infty$ as $\lambda \rightarrow + \infty$. If $\varphi ^+(-\sigma ) < 0$, then $q(-\sigma ,\delta ) < 0$ for all $\delta$, hence there is at least one real root to the right of $-\sigma$, i.e. a dominant real zero. The root is unique if, for all $\lambda \in [-\sigma , \infty )$, $q'(\lambda ,\delta )>0$ for all $\delta$, which is implied by $q'(\lambda , {\hat{\delta }}) > 0$ for all ${\hat{\delta }} \in {\hat{\mathcal {D}}}$ due to the multi-affinity of the derivative. $\square$

The presence of at least one dominant real zero suggests that there is adaptation, but is not enough: the dominant real zero must be unique in view of Proposition 5. We need therefore to establish *robust uniqueness*, again through a vertex result.

### Proposition 9

(Checking robust root uniqueness.) Let $[c^L,c^R]$ be an interval where $\varphi ^-(\lambda )$ and $\varphi ^+(\lambda )$ have opposite sign and $\varphi ^+(c^L)=0$ and $\varphi ^-(c^R)=0$ (or the other way round). Each point in the interval is a root of $q(s,\delta )$ for some $\delta \in {\mathcal {D}}$. The root is unique for all $\delta \in {\mathcal {D}}$ if all the vertex derivative polynomials $q'(s,\delta )$, $\delta \in {\hat{\mathcal {D}}}$, have the same sign (either positive or negative) in this interval. $\square$

### Proof

The intersection of $q(s,\delta )$ with the interval $[c^L,c^R]$ is unique for all $\delta \in {\mathcal {D}}$ if the derivative $q'(s,\delta )$ does not change sign in the interval for all $\delta \in {\mathcal {D}}$. Since the derivative $q'(s,\delta )$ is a multiaffine function of $\delta$, its maximum and minimum value are on the extrema, $\delta \in {\hat{\mathcal {D}}}$. $\square$

An important issue, which we now briefly discuss, is the perfect or partial adaptation to non-constant signals, for instance sinusoidal signals. To achieve perfect adaptation to a sinusoidal signal, a system must satisfy the so-called *internal model principle* (Sontag 2003), as it has been highlighted in the literature (see for instance the work by Bin et al. (2021), Mucci et al. (2020) and the references therein). In our context, *perfect adaptation* to a sinusoidal signal of frequency $\omega$ is equivalent to the condition $A_\omega (\delta )\equiv 0$, namely, it is equivalent to considering (17) with $A^+_{out}(\omega ) = 0$. This condition is quite restrictive to be ensured. Hence, we can consider *adaptation*, not perfect, just by requiring that $A^+_{out}(\omega ) \le \epsilon$, where $\epsilon >0$ is a small enough tolerance parameter. An advantage of our proposed approach is that it allows us to perform the analysis on a whole assigned interval of frequencies, rather than on a fixed one.

## Case studies of biological systems

We present in this final section some case studies of biological systems, to which we apply the techniques we have illustrated in the previous sections.

### Incoherent Feed-Forward Loop with first-order enzyme reactions

The incoherent Feed-Forward Loop (i-FFL) is a circuit where the input triggers the output response and also activates a negative controller, which eventually suppresses the output. It is known that, when the input is a ramp, the i-FFL exhibits a pulse response, while it exhibits an oscillatory output in response to a periodic input (Mangan and Alon 2003; Krishnan and Floros 2019). Here, we consider an implementation of the i-FFL realised through a synthetic transcriptional circuit (Kim et al. 2014), and we investigate to what extent small periodic perturbations of the constant input can affect the output. We let the parameters of the circuit take values in a hyper-rectangle centered around the desired nominal values. This situation reflects the fact that precisely tuning the parameters of a synthetic circuit is rarely possible.

The transcriptional i-FFL designed by Kim et al. (2014) is composed of two RNA species, *a* and *b*, whose production is controlled by the common input *u* and which hybridise to form the complex *c*. Within the complex, RNase catalyses the degradation of *b*, while it poorly catalyses the degradation of *a*; for this reason, when *c* degrades, only *a*, but not *b*, is released (see Kim et al. 2014 for details). A mathematical model of the circuit is provided by Kim et al. (2014), and the same model is adopted by Krishnan and Floros (2019) (see model KI14) to examine the response of adaptive circuits to a variety of stimuli. Assuming first-order enzyme reactions for *a*, *b*, *c* and zero-order reactions for the input *u*, the circuit is described by the following ordinary differential equations:

21 $$\begin{aligned} {\left\{ \begin{array}{ll} {\dot{a}} = u -g_1(a) -g_2(a,b,c)\\ {\dot{b}} = \kappa _2 {u} - g_3(a,b)\\ {\dot{c}} = g_2(a,b,c) \end{array}\right. } \end{aligned}$$

where $g_1(a) := a$ is the degradation term, $g_2(a,b,c) := \kappa _1(ab-c)$ describes hybridisation of *a* and *b* and the release of free *a* upon degradation of *c*, $g_3(a,b) = \kappa _2ab$ describes hybridisation of *a* and *b*. The RNA species *b* represents the output of the circuit.

When the input is a constant signal $u(t) \equiv {{\bar{u}}}$, the circuit reaches the unique equilibrium $(a_{eq},b_{eq},c_{eq}) = ({{\bar{u}}},1,{{\bar{u}}})$. Around the equilibrium, the dynamics of the circuit is described by the linearised system:

$$\begin{aligned} {\left\{ \begin{array}{ll} {\dot{\mathbf{x}}}(t) = J \mathbf{x}(t) + Ev(t)\\ y(t) = H\mathbf{x}(t) \end{array}\right. } \end{aligned}$$

where $$\mathbf{x} := \begin{bmatrix}a&b&c\end{bmatrix}^\top $$, $$E := \begin{bmatrix}1&\kappa _2&0\end{bmatrix}^\top $$, $$H := \begin{bmatrix}0&1&0\end{bmatrix}$$, and *J* is the Jacobian at the equilibrium, i.e. $J = J(a_{eq},b_{eq},c_{eq})$ with

$$\begin{aligned} J(a,b,c) = \begin{bmatrix}-1-\kappa _1b~ &{} ~-\kappa _1a~ &{} ~\kappa _1\\ -\kappa _2b &{} -\kappa _2a &{} 0\\ \kappa _1b &{} \kappa _1a &{} -\kappa _1\end{bmatrix}. \end{aligned}$$

With respect to the partial derivatives of the functions $g_1$, $g_2$ and $g_3$, the Jacobian *J*(*a*, *b*, *c*) admits the *BDC*-decomposition:

$$\begin{aligned} J(a,b,c) = \begin{bmatrix}-1~ &{} -1~ &{} -1~ &{} -1~ &{} 0~ &{} 0~\\ 0 &{} 0 &{} 0 &{} 0 &{} -1 &{} -1\\ 1 &{} 1 &{} 1 &{} 0 &{} 0 &{} 0\end{bmatrix} \begin{bmatrix}~\alpha ~ &{} ~0~ &{} ~0~ &{} ~0~ &{} ~0~ &{} ~0~\\ 0 &{} \beta &{} 0 &{} 0 &{} 0 &{} 0\\ 0 &{} 0 &{} \gamma &{} 0 &{} 0 &{} 0\\ 0 &{} 0 &{} 0 &{} \rho &{} 0 &{} 0\\ 0 &{} 0 &{} 0 &{} 0 &{} \varepsilon &{} 0\\ 0 &{} 0 &{} 0 &{} 0 &{} 0 &{} \eta \end{bmatrix} \begin{bmatrix}~1~ &{} ~0~ &{} ~0~\\ 0 &{} 1 &{} 0\\ 0 &{} 0 &{} -1\\ 1 &{} 0 &{} 0\\ 1 &{} 0 &{} 0\\ 0 &{} 1 &{} 0 \end{bmatrix} \end{aligned}$$

where:

$$\begin{aligned} \alpha :&= \frac{\partial g_2}{\partial a} = \kappa _1b&\beta :&= \frac{\partial g_2}{\partial b} = \kappa _1a&\gamma :&= \left|\frac{\partial g_2}{\partial c}\right|= \left|-\kappa _1 \right|= \kappa _1\\ \rho :&= \frac{\partial g_1}{\partial a} = 1&\varepsilon :&= \frac{\partial g_3}{\partial a} = \kappa _2b&\eta :&= \frac{\partial g_3}{\partial b} = \kappa _2a \end{aligned}$$

Let the nominal values of the parameters be $\kappa _1 = 0.01$ and $\kappa _2 = 200$, as reported in the Supplementary Material of the work by Krishnan and Floros (2019), and let the nominal input be ${{\bar{u}}} = 0.1$. Assume uncertainty bounds of $\pm 50\%$ on $\kappa _1$, $\kappa _2$. Then, the vector $\delta$ that collects the diagonal entries of matrix *D*, i.e. $$\delta = \begin{bmatrix}\alpha&\beta&\gamma&\rho&\varepsilon&\eta \end{bmatrix}^\top $$, belongs to the hyper-rectangle $$\begin{bmatrix} \delta ^-,\delta ^+\end{bmatrix}$$, where

$$\begin{aligned} \delta ^- :&= \begin{bmatrix}0.005&0.0005&0.005&1&100&10\end{bmatrix}^\top ,\\ \delta ^+ :&= \begin{bmatrix}0.015&0.0015&0.015&1&300&30\end{bmatrix}^\top . \end{aligned}$$

Note that the fourth element of $\delta$, i.e. $\rho$, inherently has value 1 because, by definition, $\rho := \frac{\partial g_1}{\partial a}$ and $\frac{\partial g_1}{\partial a} = 1$ because the model variables are non-dimensional (see the work by Kim et al. (2014) for details). Finally, let $v(t) = \mu \cos (\omega t)$ be a small periodic perturbation acting on the input ${\bar{u}}$.

**Fig. 3:**
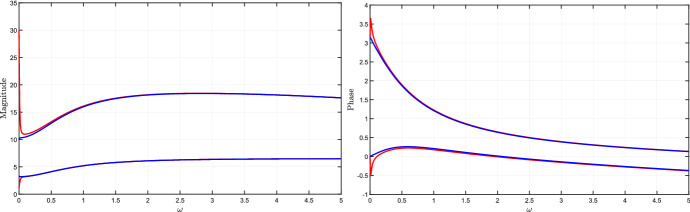
Bounds on the Bode plots of magnitude and phase of the transfer function of the i-FFL model (21). Red curves represent outer bounds (see Theorem 5 and Definition 3), while blue curves represent inner bounds (see Proposition 4) (color figure online)

By exploiting the results of Sect. 4, we can examine how, depending on the perturbation frequency $\omega$, the output *b* is affected by the perturbation *v*. Figure 3 shows lower and upper bounds on the Bode plots of the transfer function $W(i\omega ,\delta ) = H\left( i\omega I-J\right) ^{-1}E$: these bounds have been computed as in Theorem 5 and Proposition 4, and indicate, for each frequency $\omega$, the admissible values of the magnitude amplification factor and the phase shift of the output *b*.

Now assume uncertainty bounds of $\pm 50\%$ on the nominal value of the input ${\bar{u}}$, too. With respect to $\kappa _1$, $\kappa _2$ and ${\bar{u}}$, the Jacobian at the equilibrium

$$\begin{aligned} J = J(a_{eq},b_{eq},c_{eq}) = \begin{bmatrix} -1-\kappa _1 &{} -\kappa _1{{\bar{u}}} &{} \kappa _1\\ -\kappa _2 &{} -\kappa _2{{\bar{u}}} &{} 0\\ \kappa _1 &{} \kappa _1{{\bar{u}}} &{} -\kappa _1\end{bmatrix} \end{aligned}$$

is not *BDC*-decomposable.

However, since *J* is totally multiaffine in $\kappa _1$, $\kappa _2$ and ${\bar{u}}$, the vertex arguments and the results of Sect. 4 can still be applied. Let the amplitude and the frequency of the periodic perturbation *v* be $\mu = 0.05$ and $\omega _1 = 0.5$, respectively, so that $v(t) = 0.05\cos (0.5 t)$.

The computation of the numerator and denominator polynomials of the transfer function $W(s) = H\left( sI-J\right) ^{-1}E = q(s) / p(s)$ yields:

22 $$\begin{aligned} q\left( s,\kappa _1,\kappa _2\right) :&= \kappa _2s^2 + 2\kappa _1\kappa _2s \end{aligned}$$

23 $$\begin{aligned} p\left( s,\kappa _1,\kappa _2,{{\bar{u}}}\right) :&= s^3 + \left( 1+2\kappa _1+\kappa _2{{\bar{u}}}\right) s^2 + \left( \kappa _1 + \kappa _2{{\bar{u}}} + \kappa _1\kappa _2{{\bar{u}}}\right) s + \kappa _1\kappa _2{{\bar{u}}} \end{aligned}$$

**Fig. 4:**
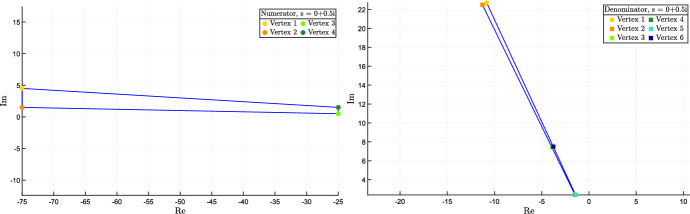
Left panel: polygon ${{\mathcal {Q}}}(i \omega _1)$ for the numerator polynomial (22) of the transfer function of the i-FFL model (21). Right panel: polygon ${{\mathcal {P}}}(i \omega _1)$ for the denominator polynomial (23) of the transfer function of the i-FFL model (21) (color figure online)

Plotting of the polygons ${{\mathcal {Q}}}(i \omega _1)$ and ${{\mathcal {P}}}(i \omega _1)$, which are reported in Fig. 4, shows that neither ${{\mathcal {Q}}}(i \omega _1)$ nor ${{\mathcal {P}}}(i \omega _1)$ includes the origin, and hence $s=i \omega _1$ is both a numerator and denominator non-critical value. Then, bounds on magnitude and phase of each polynomial can be computed as in Theorem 4, which yields:

$$\begin{aligned} \theta _q^-&= 3.0817&\theta _q^+&= 3.1216&\mu _q^+&= 75.1349&\mu _q^-&= 25.0050\\ \theta _p^-&= 2.0157&\theta _p^+&= 2.1245&\mu _p^+&= 25.1702&\mu _p^-&= 2.8098 \end{aligned}$$

Finally, in view of Theorem 5, the amplification factor $A_\omega$ and the phase shift $\phi _\omega$ of the output *b* satisfy the following inequalities:

24 $$\begin{aligned} \frac{\mu ^-_q}{\mu ^+_p} = 0.9934 \le&A_\omega \le 26.7403 = \frac{\mu ^+_q}{\mu ^-_p} \end{aligned}$$

25 $$\begin{aligned} \theta ^-_q - \theta ^+_p = 0.9572 \le&\phi _\omega \le 1.1059 = \theta ^+_q - \theta ^-_p \end{aligned}$$

**Fig. 5:**
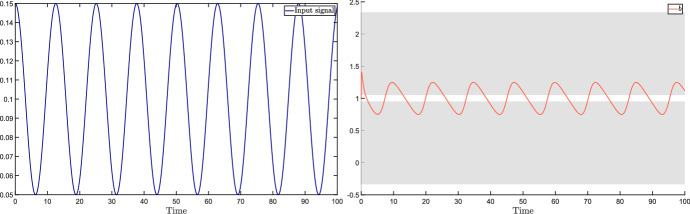
Left panel: input signal ${{\bar{u}}} + v(t)$. The periodic perturbation is $v(t) = 0.05\cos (\omega _1 t)$ with $\omega _1 = 0.5$. Right panel: output *b*(*t*). The gray area indicates the interval between the maximum and minimum values of the output allowed by the bounds (24) (color figure online)

Figure 5 reports the perturbed input signal ${{\bar{u}}} + v(t)$ and the output *b*(*t*). The interval between the maximum and minimum values of the output allowed by the bounds (24) is indicated by the gray area: as it can be seen, the bounds on the output amplitude are indeed satisfied.

**Fig. 6:**
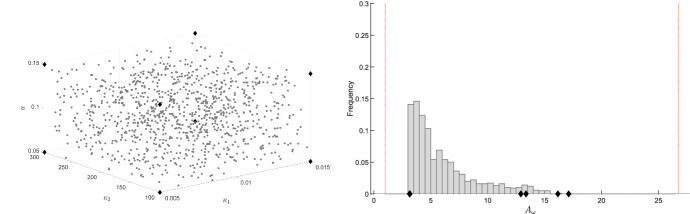
Left panel: $N=1000$ sets of values for $\kappa _1$, $\kappa _2$ and ${\bar{u}}$, randomly sampled within the hyper-rectangle whose vertices are indicated by the black diamonds. Right panel: histogram of the output amplification factors corresponding to the randomly sampled parameters. Black diamonds indicate the amplification factors corresponding to the vertices of the hyper-rectangle. Red dashed lines indicate the bounds (24) (color figure online)

To investigate the degree of conservatism of the bounds (24), $N = 1000$ sets of parameters have been randomly sampled within the given hyper-rectangle and, for each set of parameters, the amplification factor of the output has been computed. Figure 6 shows the parameters sampled within the hyper-rectangle (left panel) and a histogram of the amplification factors (right panel), where the red dashed lines indicate the bounds (24). Black diamonds indicate the vertices of the hyper-rectangle (left panel) and the corresponding amplification factors (right panel).

### Coherent Feed-Forward Loop with Hill dynamics

In a coherent Feed-Forward Loop (c-FFL), activation of the most downstream element $x_3$ of the circuit occurs both directly from the most upstream element $x_1$ and indirectly, via the intermediate element $x_2$, which is, in turn, activated by $x_1$. A c-FFL with Hill-type dynamics is described by the following set of equations (see the work by Ghafari and Mashaghi (2017)):

26 $$\begin{aligned} {\left\{ \begin{array}{ll} {\dot{x}}_1 &{}= u - g_1(x_1)\\ {\dot{x}}_2 &{}= {{\mathcal {H}}}_1^+(x_1) - g_2(x_2)\\ {\dot{x}}_3 &{}= {{\mathcal {H}}}_2^+(x_2) \cdot {{\mathcal {H}}}_1^+(x_1) - g_3(x_3) \end{array}\right. } \end{aligned}$$

where *u* is the external input, ${{\mathcal {H}}}_i^+(x_i) = V_i \frac{x_i^n}{x_i^n+K_{iA}^n}$, $i=1,2$, is the activating Hill function, and $g_i(x_i) := \gamma _i x_i$, $i=1,2,3$ are the degradation terms. The downstream element $x_3$ is taken as output of the circuit.

When the input is a constant signal $u(t) \equiv {{\bar{u}}}$, the circuit reaches the unique equilibrium:

$$\begin{aligned} x_{1eq} = \frac{{\bar{u}}}{\gamma _1}, \qquad x_{2eq} = \frac{{\mathcal H}_1^+(x_{1eq})}{\gamma _2}, \qquad x_{3eq} = \frac{{\mathcal H}_2^+(x_{2eq}) {{\mathcal {H}}}_1^+(x_{1eq})}{\gamma _3} \end{aligned}$$

Around the equilibrium, the dynamics of the circuit is described by the linearised system

$$\begin{aligned} {\left\{ \begin{array}{ll} {\dot{\mathbf{x}}}(t) = J\mathbf{x}(t) + Ev(t)\\ y(t) = H\mathbf{x}(t) \end{array}\right. } \end{aligned}$$

where $$\mathbf{x} := \begin{bmatrix}x_1&x_2&x_3\end{bmatrix}^\top $$, $$E := \begin{bmatrix}1&0&0\end{bmatrix}^\top $$, $$H := \begin{bmatrix}0&0&1\end{bmatrix}$$, and the Jacobian is given by

$$\begin{aligned} J := J(x_{1eq},x_{2eq},x_{3eq}) = \begin{bmatrix}-\gamma _1 &{} 0 &{} 0\\ a_1(x_{1eq}) &{} -\gamma _2 &{} 0\\ {{\mathcal {H}}}_2^+(x_{2eq})a_1(x_{1eq}) &{} {{\mathcal {H}}}_1^+(x_{1eq})a_2(x_{2eq}) &{} -\gamma _3\end{bmatrix} \end{aligned}$$

where we have set:

$$\begin{aligned} a_i(x_i) := \frac{\partial {{\mathcal {H}}}_i^+(x_i)}{\partial x_i} = n V_i K_{iA}^n \frac{x_i^{n-1}}{(x_i^n + K_{iA}^n)^2}, \qquad i=1,2 \end{aligned}$$

Upon definig $\alpha := a_1(x_{1eq})$, $\beta := {\mathcal H}_1^+(x_{1eq})a_2(x_{2eq})$ and $\varepsilon := {\mathcal H}_2^+(x_{2eq})a_1(x_{1eq})$, the Jacobian admits the *BDC*-decomposition:

$$\begin{aligned} J = \begin{bmatrix} -1 &{} 0 &{} 0 &{} 0 &{} 0 &{} 0\\ 0 &{} -1 &{} 0 &{} 1 &{} 0 &{} 0\\ 0 &{} 0 &{} -1 &{} 0 &{} 1 &{} 1 \end{bmatrix} \begin{bmatrix} \gamma _1 &{} 0 &{} 0 &{} 0 &{} 0 &{} 0\\ 0 &{} \gamma _2 &{} 0 &{} 0 &{} 0 &{} 0\\ 0 &{} 0 &{} \gamma _3 &{} 0 &{} 0 &{} 0\\ 0 &{} 0 &{} 0 &{} \alpha &{} 0 &{} 0\\ 0 &{} 0 &{} 0 &{} 0 &{} \beta &{} 0\\ 0 &{} 0 &{} 0 &{} 0 &{} 0 &{} \varepsilon \end{bmatrix} \begin{bmatrix} 1 &{} 0 &{} 0\\ 0 &{} 1 &{} 0\\ 0 &{} 0 &{} 1\\ 1 &{} 0 &{} 0\\ 0 &{} 1 &{} 0\\ 1 &{} 0 &{} 0\end{bmatrix} \end{aligned}$$

Since *J* is *BDC*-decomposable, it is multi-affine in $\gamma _1$, $\gamma _2$, $\gamma _3$, $\alpha$, $\beta$ and $\varepsilon$. Indeed, computation of the transfer function $W(s) = H\left( sI-J\right) ^{-1}E = q(s) / p(s)$ shows that both numerator and denominator polynomials are multi-affine:

27 $$\begin{aligned} q(s,\gamma _2,\alpha ,\beta ,\varepsilon ) :&= \varepsilon s + \alpha \beta + \delta \gamma _2 \end{aligned}$$

28 $$\begin{aligned} p(s,\gamma _1,\gamma _2,\gamma _3) :&= (s+\gamma _1)(s+\gamma _2)(s+\gamma _3) \end{aligned}$$

Let the nominal values of the parameters be $\gamma _1 = \gamma _2 = \gamma _3 = 1$, $V_1 = 0.6$, $V_2 = 0.5$, $K_{1A} = K_{2A} = 1$, $n = 2$ (biologically plausible values are provided e.g. by Ghafari and Mashaghi 2017; Smolen et al. 2000; Mangan and Alon 2003), and let the nominal input be ${{\bar{u}}} = 1$. Then, the nominal vector $\delta$ that collects the diagonal entries of matrix *D* is $$\delta = \begin{bmatrix}\gamma _1&\gamma _2&\gamma _3&\alpha&\beta&\varepsilon \end{bmatrix}^\top = \begin{bmatrix}1&1&1&0.3&0.0758&0.0124\end{bmatrix}^\top $$. Assume uncertainty bounds equal to $\pm 50\%$ on the entries of $\delta$, namely assume that $\delta$ belongs to the hyper-rectangle $$\begin{bmatrix} \delta ^-,\delta ^+\end{bmatrix}$$, where

$$\begin{aligned} \delta ^- :&= \begin{bmatrix}0.5&0.5&0.5&0.15&0.0379&0.0062\end{bmatrix}^\top \\ \delta ^+ :&= \begin{bmatrix}1.5&1.5&1.5&0.45&0.1136&0.0186\end{bmatrix}^\top \end{aligned}$$

**Fig. 7:**
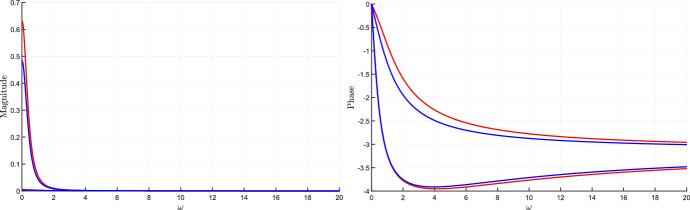
Bounds on the Bode plots of the transfer function of the c-FFL model (26). Red curves represent outer bounds (see Theorem 5 and Definition 3), while blue curves represent inner bounds (see Proposition 4) (color figure online)

Let $v(t) = \mu \cos (\omega t)$ be a small periodic perturbation acting on the input ${\bar{u}}$. By applying the results of Sect. 4, we can examine the effects of the periodic perturbation *v* on the output $x_3$. The bounds on the Bode plots of the transfer function $W(i\omega ,\delta ) = H\left( i\omega I-J\right) ^{-1}E$ are reported in Fig. 7: they provide, for each perturbation frequency $\omega$, admissible intervals for the amplification factor and the phase shift of the output $x_3$.

Now let the amplitude and the frequency of the periodic perturbation *v* be $\mu = 0.05$ and $\omega _1 = 0.55$, respectively, namely let $v(t) = 0.05\cos (0.55t)$. We investigate, in two different scenarios, the degree of conservatism of the bounds on the amplification factor.

**Fig. 8:**
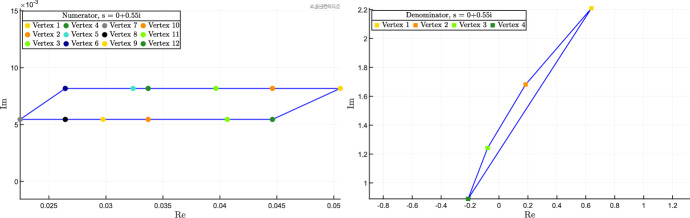
Left panel: polygon ${{\mathcal {Q}}}(i \omega _1)$ for the numerator polynomial (27) of the transfer function of the c-FFL model (26). Right panel: polygon ${{\mathcal {P}}}(i \omega _1)$ for the denominator polynomial (28) of the transfer function of the c-FFL model (26) (color figure online)

**Fig. 9:**
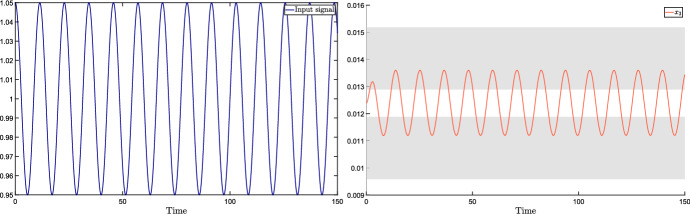
Left panel: input signal ${{\bar{u}}} + v(t)$. The periodic perturbation is $v(t) = 0.05\cos (\omega _1 t)$ with $\omega _1 = 0.55$. Right panel: output $x_3(t)$. The gray area indicates the interval between the maximum and minimum values of the output allowed by the bounds computed as in Theorem 4 and Theorem 5 (color figure online)

**Fig. 10:**
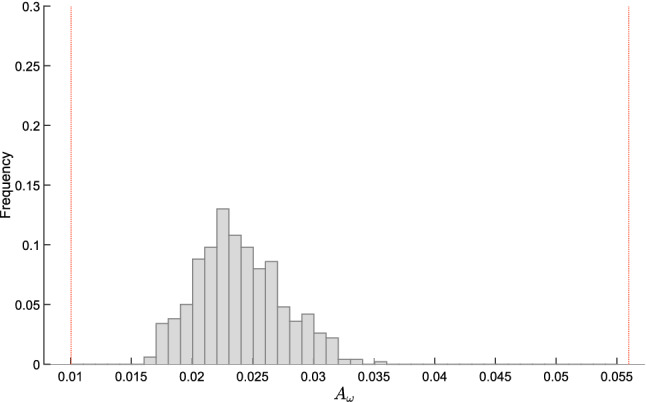
Histogram of the output amplification factors corresponding to $N=500$ sets of parameters $\gamma _1$, $\gamma _2$, $\gamma _3$, $V_1$, $V_2$, $K_{1A}$, $K_{2A}$, for which the bounds of $\pm 20\%$ on the entries of $\delta$ are satisfied. Red dashed lines indicate the bounds computed as in Theorem 4 and Theorem 5 (color figure online)

In the first scenario, we assume uncertainty bounds of $\pm 20\%$ on all entries of the vector $\delta$. Plotting of the polygons ${{\mathcal {Q}}}(i \omega _1)$ and ${{\mathcal {P}}}(i \omega _1)$, which are reported in Fig. 8, shows that neither ${{\mathcal {Q}}}(i \omega _1)$ nor ${{\mathcal {P}}}(i \omega _1)$ includes the origin, and hence $s=i \omega _1$ is both a numerator and denominator non-critical value. Then, bounds on the amplification factor of the output $x_3$ can be computed by exploiting Theorem 4 and Theorem 5. Figure 9 reports the perturbed input signal ${{\bar{u}}} + v(t)$ and the output $x_3(t)$ together with the interval values allowed by the bounds. To investigate the degree of conservatism of the computed bounds, we randomly sample the parameters $\gamma _1$, $\gamma _2$, $\gamma _3$, $V_1$, $V_2$, $K_{1A}$, $K_{2A}$, and we retain $N = 500$ sets of parameters for which the bounds of $\pm 20\%$ on the entries of $\delta$ are satisfied. Then, for each set of parameters, we compute the amplification factor of the simulated output. A histogram of the amplification factors is reported in Fig. 10.

**Fig. 11:**
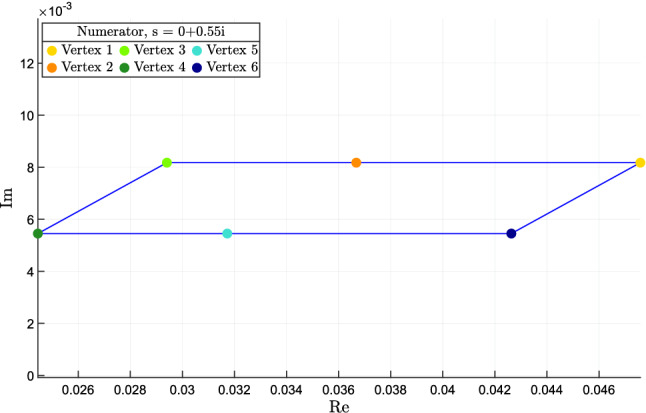
Polygon ${{\mathcal {Q}}}(i \omega _1)$ for the numerator polynomial (27) of the transfer function of the c-FFL model (26) (color figure online)

**Fig. 12:**
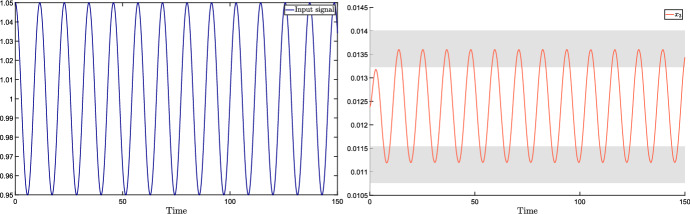
Left panel: input signal ${{\bar{u}}} + v(t)$. The periodic perturbation is $v(t) = 0.05\cos (\omega _1 t)$ with $\omega _1 = 0.55$. Right panel: output $x_3(t)$. The gray area indicates the interval between the maximum and minimum values of the output allowed by the bounds computed as in Theorem 4 and Theorem 5 (color figure online)

**Fig. 13:**
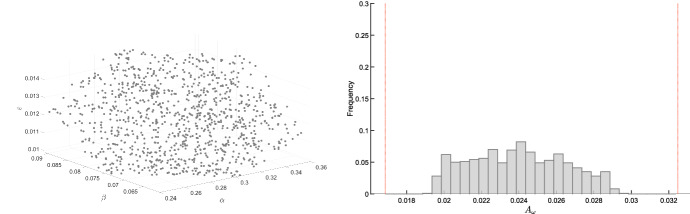
Left panel: values of $\alpha$, $\beta$ and $\varepsilon$ for $N=500$ randomly sampled values of $V_1$, $V_2$ and $K_{1A} = K_{2A}$. Right panel: histogram of the values of the magnitude amplification factor on the simulated output $x_3$ (color figure online)

In the second scenario, we fix $\gamma _1$, $\gamma _2$, and $\gamma _3$ to the nominal values, and we assume bounds of $\pm 20\%$ on entries $\alpha$, $\beta$, and $\varepsilon$ of the vector $\delta$. In this case, the denominator polynomial $p(s,\gamma _1,\gamma _2,\gamma _3)$ is not subject to uncertainty. Plotting of the polygon ${{\mathcal {Q}}}(i \omega _1)$, which is reported in Fig. 11, shows that ${{\mathcal {Q}}}(i \omega _1)$ does not include the origin, and hence $s=i \omega _1$ is both a numerator and denominator non-critical value. Then, bounds on the amplification factor of the output $x_3$ can be computed by exploiting Theorem 4 and Theorem 5. Figure 12 reports the perturbed input signal ${{\bar{u}}} + v(t)$ and the output $x_3(t)$ together with the computed bounds (note that, with respect to Fig. 9, the input and the output signals are the same, while the bounds are different). It can be seen that the output stays within the gray area. To investigate the degree of conservatism, we randomly sample the parameters $V_1$, $V_2$, $K_{1A}$, and $K_{2A}$, we retain $N = 1000$ sets of parameters for which the bounds of $\pm 20\%$ on $\alpha$, $\beta$, and $\varepsilon$ are satisfied, and we compute, for each set of parameters, the amplification factor of the simulated output. Figure 13 shows the values of $\alpha$, $\beta$, and $\varepsilon$ for the retained sets of sampled parameters and a histogram of the amplification factors.

### The Brusselator oscillator

Consider the well-known Brusselator oscillator as described for instance by Epstein and Pojman (1998). The model consists of the following two differential equations:

29 $$\begin{aligned} {\left\{ \begin{array}{ll} \dot{x} = -a x +bx^2y - cx +u\\ \dot{y} = +a x -bx^2y \end{array}\right. } \end{aligned}$$

The Jacobian of the system is:

$$\begin{aligned} J(a,b,c) = \begin{bmatrix} -a + 2bxy-c &{} bx^2 \\ +a - 2bxy &{} - bx^2 \end{bmatrix} . \end{aligned}$$

At the equilibrium, it holds $a= bx_{eq}y_{eq}$ and the Jacobian is therefore:

$$\begin{aligned} J = \begin{bmatrix} a-c &{} bx_{eq}^2 \\ -a &{} - bx_{eq}^2 \end{bmatrix}. \end{aligned}$$

**Fig. 14:**
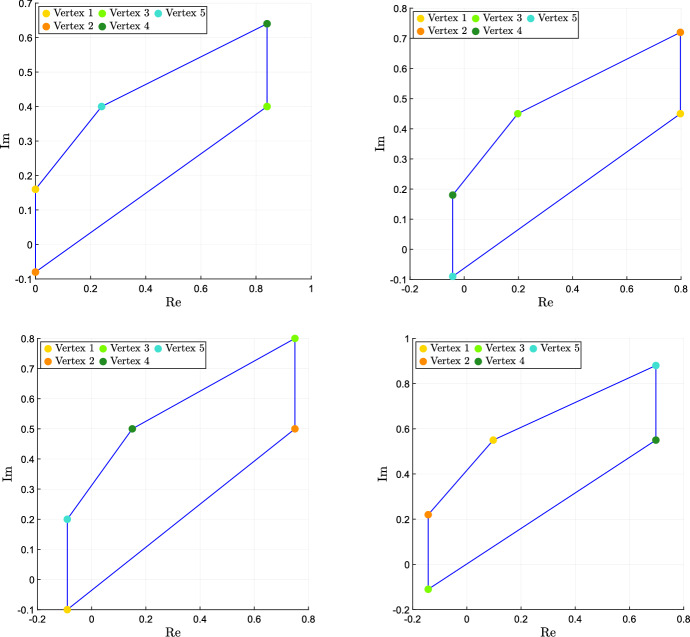
Polygons ${\mathcal {P}}(i \omega )$ for the characteristic polynomial $p(s,\delta _1,\delta _2,\delta _3)$ of the Brusselator model (29). The frequency is $\omega =0.4$ (upper, left panel), $\omega =0.45$ (upper, right panel), $\omega =0.5$ (bottom, left panel), and $\omega =0.55$ (bottom, right panel). Parameters values: $a=0.95$, $b=0.5$, $c=0.4$ $u=0.4$ (color figure online)

To perform our analysis, we re-parameterise the problem adopting the parameters $\delta _1=a$, $\delta _2=c$ and $\delta _3=bx_{eq}^2$, on which we impose the uncertainty bounds. The Jacobian can thus be written as:

$$\begin{aligned} J = \begin{bmatrix} \delta _1-\delta _2 &{} \delta _3\\ -\delta _1 &{} -\delta _3 \end{bmatrix}, \end{aligned}$$

whose characteristic polynomial is $p(s,\delta _1,\delta _2,\delta _3) = s^2 + (-\delta _1 + \delta _2 + \delta _3)s + \delta _2 \delta _3$. We assume uncertainty bounds of $0.4 \le \delta _i \le 1$, $i=1,2,3$. By applying Theorem 6, we can identify potential oscillatory frequencies of the system. To this aim, we plot some polygons ${\mathcal {P}}(i \omega )$ of the polynomial $p(s,\delta _1,\delta _2,\delta _3)$: Fig. 14 shows some polygons ${\mathcal {P}}(i \omega )$ for frequency $\omega$ in the interval [0.4, 0.55]. Since each of these polygons includes the origin, we can conclude that all frequencies $\omega$ with $0.4 \le \omega \le 0.55$ are potential oscillatory frequencies.

**Fig. 15:**
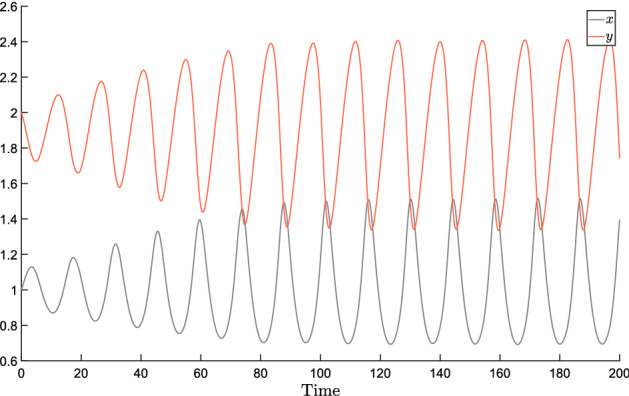
Simulation of the Brusselator model (29). The parameter values are the same as in Fig. 14 (color figure online)

To verify that the system exhibits oscillations in this range of frequencies, let us consider for numerical purposes the following data: $a=0.95$, $b=0.5$, $c=0.4$ (reaction rate constants) and $u=0.4$ (input). These are proper parameters for the model; to perform our analysis, we consider the parameters $\delta _i$, on which the uncertainty bounds are imposed. With these values of the original parameters, the bounds $0.4 \le \delta _i \le 1$ are satisfied; indeed, it results: $\delta _1 = 0.95$, $\delta _2=0.4$ and $\delta _3=0.5$. Simulation of the model, reported in Fig. 15, shows that, with this set of parameters, the system exhibits sustained oscillations, and the frequency of oscillation is about $\omega \approx 0.44$, consistently with the fact that the polygon ${\mathcal {P}}(i 0.44)$ includes the origin.

### The Goldbeter oscillator

The Brusselator was a very simple, illustrative example of oscillator. In this section, we consider a more complex oscillator, which describes the circadian oscillations of PER protein in *Drosophila*. The system is based on multiple phosphorylation of PER and on the negative feedback implemented by the nuclear form of PER on the transcription of the *per* gene. A mathematical description of the system was proposed by Goldbeter (1995). The Goldbeter oscillator model includes five state variables: *M* denotes the concentration of mRNA transcripts from *per* gene; $P_0$, $P_1$ and $P_2$ denote the concentration of PER protein in the unphosphorylated, monphosphorylated and biphosphorylated form, respectively; $P_N$ denotes the concentration of the nuclear form of PER. The system is described by the following five differential equations:

30 $$\begin{aligned} {\left\{ \begin{array}{ll} \dot{M} &{}= v_s \frac{K_I^n}{K_I^n+P_N^n} - v_m \frac{M}{K_m+M}\\ \dot{P}_0 &{}= k_s M - V_1 \frac{P_0}{K_1+P_0} + V_2 \frac{P_1}{K_2+P_1}\\ \dot{P}_1 &{}= {V_1} \frac{P_0}{K_1+P_0} - V_2 \frac{P_1}{{K_2} +P_1} -V_3\frac{P_1}{K_3+P_1} + V_4 \frac{P_2}{K_4+P_2}\\ \dot{P}_2 &{}= V_3\frac{P_1}{K_3+P_1} - V_4 \frac{P_2}{K_4+P_2} - k_1 P_2 { + k_2 P_N} - v_d\frac{P_2}{K_d+P_2}\\ \dot{P}_N &{}= k_1 P_2 - k_2 P_N \end{array}\right. } \end{aligned}$$

The total concentration of PER protein, denoted by $P_t$, is computed as $P_t := P_0 + P_1 + P_2 + P_N$.

Straightforward computations show that the Jacobian of the system takes the following form:

31 $$\begin{aligned} J = \begin{bmatrix} ~~-\alpha ~~ &{} ~~0~~ &{} ~~0~~ &{}~~0~~ &{}~~-\rho ~~\\ k_s &{} -\gamma &{} \beta &{} 0 &{}0 \\ 0 &{} \gamma &{} -(\beta +\varepsilon ) &{} \phi &{} 0\\ 0 &{} 0 &{}\varepsilon &{} -(\phi +\nu +k_1) &{} k_2\\ 0 &{} 0 &{} 0 &{}k_1 &{} -k_2 \end{bmatrix} \end{aligned}$$

where the Greek letters denote the partial derivatives of the Michaelis-Menten and Hill functions, specifically:

$$\begin{aligned} \alpha :&= \frac{v_m K_m}{(K_m + M)^2}&\gamma :&= \frac{V_1 K_1}{(K_1 + P_0)^2}&\beta :&= \frac{V_2 K_2}{(K_2 + P_1)^2}&\varepsilon :&= \frac{V_3 K_3}{(K_3 + P_1)^2}\\ \phi :&= \frac{V_4 K_4}{(K_4 + P_2)^2}&\nu :&= \frac{v_d K_d}{(K_d + P_2)^2}&\rho :&= \frac{n v_s K_I^n P_N^{n-1}}{(K_I^n + P_N^n)^2}&\end{aligned}$$

Since the Jacobian is multi-affine in $\alpha$, $\beta$, $\gamma$, $\varepsilon$, $\phi$, $\nu$, $\rho$, $k_s$, $k_1$, $k_2$, we can apply the results of Sect. 4.2 to identify potential oscillatory frequencies. Let the nominal values of the parameters be the values adopted by Goldbeter (1995), namely: $v_s=0.76 \mu M h^{-1}$, $v_m=0.65 \mu M h^{-1}$, $K_m=0.5 \mu M$, $k_s=0.38 h^{-1}$, $v_d=0.95 \mu M h^{-1}$, $k_1=1.9 h^{-1}$, $k_2=1.3 h^{-1}$, $K_d=0.2 \mu M$, $n=4$, $K_I=1 \mu M$, $K_1=2 \mu M$, $K_2=2 \mu M$, $K_3=2 \mu M$, $K_4=2 \mu M$, $V_1=3.2 \mu M h^{-1}$, $V_2=1.58 \mu M h^{-1}$, $V_3=5 \mu M h^{-1}$, $V_4=2.5 \mu M h^{-1}$. Consider as working point of the system the rough values around which *M*, $P_0$, $P_1$, $P_2$ and $P_N$ are known to oscillate (Goldbeter 1995), namely: ${\bar{M}} = 1.75 \mu M$, $\bar{P}_0 = 1.1 \mu M$, ${\bar{P}}_1 = 0.67 \mu M$, ${\bar{P}}_2 = 0.58 \mu M$, ${\bar{P}}_N = 0.85 \mu M$. For the nominal values of the parameters and the considered working point, the Jacobian is:

$$\begin{aligned} {\bar{J}} = \begin{bmatrix} -0.06 &{} 0 &{} 0 &{} 0 &{} -0.81 \\ 0.38 &{} -0.68 &{} 0.44 &{} 0 &{} 0\\ 0 &{} 0.68 &{} -1.85 &{} 0.75 &{} 0\\ 0 &{} 0 &{} 1.41 &{} -2.96 &{} 1.3\\ 0 &{} 0 &{} 0 &{} 1.9 &{} -1.3 \end{bmatrix} \end{aligned}$$

**Fig. 16:**
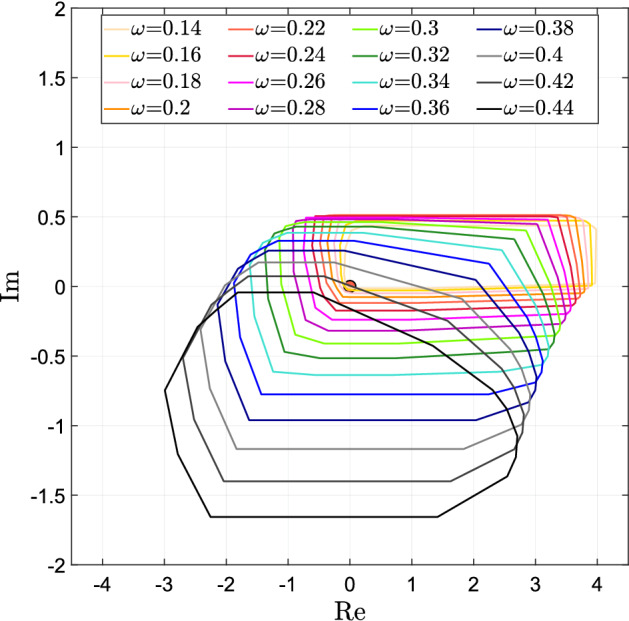
Polygons ${\mathcal {P}}(i\omega )$ of the characteristic polynomial of the Jacobian (31) for frequencies $\omega$ in the interval $[\omega _{min},\omega _{max}] = [0.14,0.44]$ (color figure online)

**Fig. 17:**
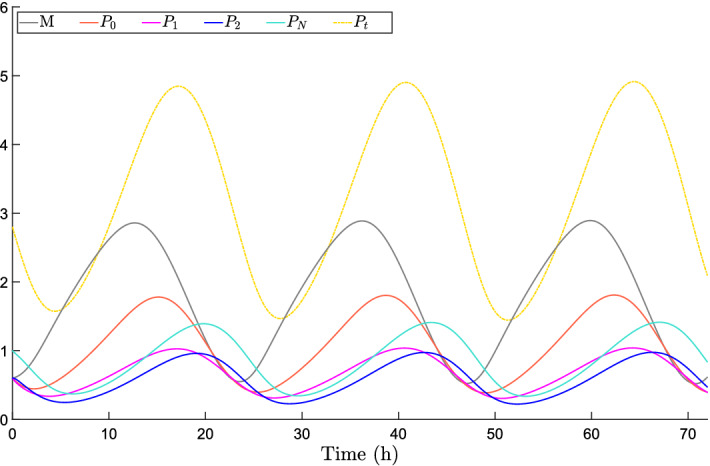
Dynamic evolution of the *Drosophila* oscillator model (30). Numerical values of the parameters are the nominal ones adopted by Goldbeter (1995) (color figure online)

Assume rough uncertainty bounds of $\pm 50 \%$ on each non-zero entry of the Jacobian, namely on $\alpha$, $\beta$, $\gamma$, $\varepsilon$, $\phi$, $\nu$, $\rho$, $k_s$, $k_1$, $k_2$. To identify potential oscillatory frequencies of the system, we compute the characteristic polynomial of the Jacobian (31), and we plot the polygon $\mathcal P(i\omega )$, which is the convex hull of the value set, in a large range of frequencies. As seen from Fig. 16, ${\mathcal {P}}(i\omega )$ includes the origin for frequencies $\omega$ in the interval $[\omega _{min},\omega _{max}] = [0.14,0.42]$. These frequencies represent potential oscillatory frequencies of the system. The corresponding oscillation period ranges from $T_{min} = 2\pi / \omega _{max} = 14.96 h$ to $T_{max} = 2\pi / \omega _{min} = 44.88 h$. As reported in Fig. 17, this is fully consistent with the numerical simulations proposed by Goldbeter (1995).

### Adaptation analysis

The biochemical reaction networkcan be associated with a system of differential equations, describing the time evolution of the species concentrations, having state vector $$x = \begin{bmatrix} x_1&x_2&x_3&x_4 \end{bmatrix}^\top $$:

32 $$\begin{aligned} {\left\{ \begin{array}{ll} \dot{x}_1 = k_1 - g_{12}(x_1,x_2)-g_{14}(x_1,x_4)\\ \dot{x}_2 = k_2 - g_{12}(x_1,x_2)-g_2(x_2)\\ \dot{x}_3 = g_{12}(x_1,x_2)-g_3(x_3)\\ \dot{x}_4 = g_3(x_3)-g_{14}(x_1,x_4)-g_4(x_4) \end{array}\right. } \end{aligned}$$

Linearisation yields a *BDC*-decomposable system with Jacobian $J=B D C$, where

$$\begin{aligned} B&= \begin{bmatrix} -1 &{} -1 &{} 0 &{} -1 &{} -1 &{} 0 &{} 0\\ -1 &{} -1 &{} 0 &{} 0 &{} 0 &{} -1 &{} 0\\ 1 &{} 1 &{} -1 &{} 0 &{} 0 &{} 0 &{} 0\\ 0 &{} 0&{} 1 &{} -1 &{} -1 &{} 0 &{} -1 \end{bmatrix}, \\ D&= \text {diag}\left\{ \frac{\partial g_{12}}{\partial x_1}, \frac{\partial g_{12}}{\partial x_2}, \frac{\partial g_{3}}{\partial x_3}, \frac{\partial g_{14}}{\partial x_1}, \frac{\partial g_{14}}{\partial x_4}, \frac{\partial g_{2}}{\partial x_2}, \frac{\partial g_{4}}{\partial x_4}\right\} \doteq {{\,\mathrm{diag}\,}}(\delta ),\\ C&= \begin{bmatrix} 1 &{} 0 &{} 0 &{} 1 &{} 0 &{} 0 &{} 0\\ 0 &{} 1 &{} 0 &{} 0 &{} 0 &{} 1 &{} 0\\ 0 &{} 0 &{} 1 &{} 0 &{} 0 &{} 0 &{} 0\\ 0 &{} 0 &{} 0 &{} 0 &{} 1 &{} 0 &{} 1 \end{bmatrix}. \end{aligned}$$

**Fig. 18:**
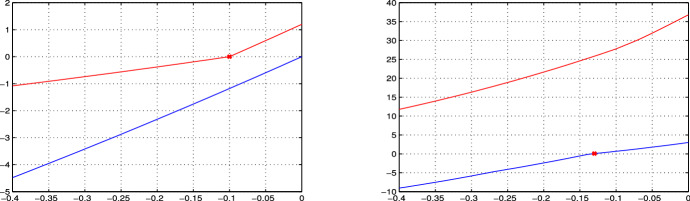
Robust real plot with lower and upper bounding functions for the numerator, left, and the denominator, right, in Example 6.5. The real pole and zero intervals are disjoint and the zero dominates the pole (color figure online)

**Fig. 19:**
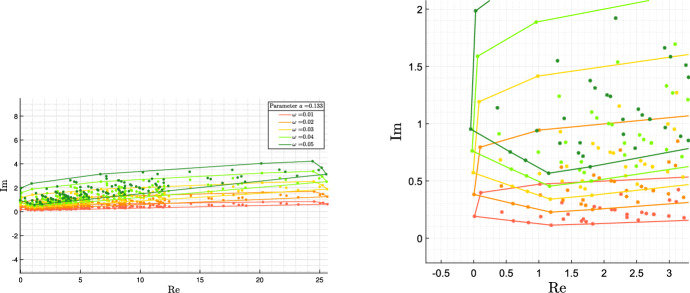
The set ${{\mathcal {P}}}(i \omega - a)$ in the example in Sect. 6.5 for $a=0.133$ and $\omega \in [0.01,0.05]$, left, along with a zoom-in panel to better visualise the neighbourhood of the origin, right (color figure online)

We consider an additive input on $x_1$, namely $E=[1~ 0~ 0~ 0]^\top$, and we take $x_3$ as output, namely $H=[0 ~0~ 1 ~0]$. The lower and upper bounds on $\delta$ are given as $\delta ^-=[1 ~1 ~1~ 1~ 1 ~0.0 ~3]$ and $\delta ^+=[2 ~2~ 2 ~2 ~2 ~0.1 ~4]$. For $\delta _6=0$, it can be seen that the system has perfect adaptation: the transfer function has a zero at the origin. We wish to assess whether adaptation (although non-perfect) is robustly maintained for small values of $\delta _6$? The robust real plot is shown in Fig. 18 and shows that the dominant zero interval $[-0.1,-0.0]$ is to the right of the dominant pole interval, $[-0.79,-0.133]$. Therefore, we have robust real zero dominance. The same conclusion can be drawn by noticing that $-a=-0.133$ is a robust spectral abscissa, since the origin does not belong to the set ${{\mathcal {P}}}(i \omega - a)$ as can be seen in Fig. 19, and that $\psi ^+(-a)<0$.

## Concluding discussion

This paper aims at building a connection between robustness analysis problems that arise in systems biology and a class of powerful robustness analysis tools developed within the realm of control theory (Barmish 1994). Although the general principles of biological robustness have been thoroughly discussed from a variety of perspectives (Khammash 2016; Kitano 2004; Lesne 2008; Stelling et al. 2004; Streif et al. 2016; Whitacre 2012) and several dedicated tools have been adopted to perform the robustness analysis of specific models, or classes of models, as discussed in the introduction, we are still lacking a general framework that allows us to robustly assess properties of interest for biological models in a systematic way.

For the first time to our knowledge, this work proposes to leverage the vertex-type results developed in the 90s in the control community (Barmish 1994) for the robustness analysis of a vast class of biological models that turn out to have a totally multiaffine uncertainty structure: the class we consider includes generic biochemical reaction networks, as well as chemical reaction networks with mass-action kinetics. For this class of systems, building upon the work by Barmish (1994), we derive vertex results that allow us to robustly assess the input-output sensitivity, in the presence of input perturbations that can be either constant or periodic, in the neighbourhood of an equilibrium state. Besides being useful for the robust steady-state sensitivity analysis in the presence of constant perturbations and for the robust frequency-response sensitivity analysis in the presence of sinusoidal disturbances, vertex-type algorithms also enable to robustly assess non-singularity, stability, and adaptation.

Our analysis has two main limitations. First, it is based on linearisation: all our characterisations do transfer from the linear approximation to the original nonlinear system when small input signals are considered, but the prediction capability may be lost for large inputs. Second, the analysis requires in general a re-parametrisation, namely, it considers uncertain parameters that may be functions of the original parameters: as a consequence, providing uncertainty bounds is not always straightforward, and a certain degree of conservatism is introduced, which we have numerically quantified in some case studies.

A benefit of the approach is that it allows us to resort to very effective methods available in the literature, which are shown to be applicable to study biologically relevant models and provide a critical insight into their robustness analysis.

A potentially interesting alternative approach to the robustness analysis of biological systems relies on the probabilistic methods and randomised algorithms introduced by Tempo et al. (2005), which tackle uncertain systems by assessing their properties in probability, through sampling (Monte Carlo) techniques. These approaches have the advantage of being quite flexible, since they do not require special structures (e.g. multiaffinity) and they can fruitfully support other methodologies, as shown for instance in the case study by Kim et al. (2006). However, they have two main drawbacks: the number of samples required to get to a conclusion with a given confidence may be huge, and their outcome is heavily distribution-dependent, so that different sample distributions for the system parameters can result in completely different conclusions on the system properties. A possible future research direction is that of systematically combining probabilistic and classic approaches for the robustness analysis of biological models.
